# Synthesis and Antiplatelet Activity of Antithrombotic Thiourea Compounds: Biological and Structure-Activity Relationship Studies

**DOI:** 10.3390/molecules20047174

**Published:** 2015-04-20

**Authors:** André Luiz Lourenço, Max Seidy Saito, Luís Eduardo Gomes Dorneles, Gil Mendes Viana, Plínio Cunha Sathler, Lúcia Cruz de Sequeira Aguiar, Marcelo de Pádula, Thaisa Francielle Souza Domingos, Aline Guerra Manssour Fraga, Carlos Rangel Rodrigues, Valeria Pereira de Sousa, Helena Carla Castro, Lucio Mendes Cabral

**Affiliations:** 1 Programa de Pós-graduação em Patologia, Departamento de Patologia, Hospital Universitário Antônio Pedro (HUAP), Universidade Federal Fluminense (UFF), Niterói CEP 24033-900, RJ, Brazil; E-Mails: andrebiouff@gmail.com (A.L.L.); maxsaito@gmail.com (M.S.S.); 2 LabTIF, Faculdade de Farmácia, Universidade Federal do Rio de Janeiro (UFRJ), Rio de Janeiro CEP 21941-902, RJ, Brazil; E-Mails: legdorneles@yahoo.com.br (L.E.G.D.); gmviana@gmail.com (G.M.V.); pliniocs@yahoo.com.br (P.C.S.); marcelo@pharma.ufrj.br (M.P.); thaisadomingos@yahoo.com.br (T.F.S.D.); agmfraga@yahoo.com.br (A.G.M.F.); valeria@pharma.ufrj.br (V.P.S.); 3 Instituto de Química, Universidade Federal do Rio de Janeiro (UFRJ), Rio de Janeiro CEP 21941-909, RJ, Brazil; E-Mail: luciasequeira@yahoo.com.br; 4 ModMolQSAR, Faculdade de Farmácia, Universidade Federal do Rio de Janeiro (UFRJ), Rio de Janeiro CEP 21941-902, RJ, Brazil; E-Mail: rangelfarmacia@gmail.com; 5 LABiEMOL, Departamento de Biologia Celular e Molecular, Universidade Federal Fluminense (UFF), Niterói CEP 24033-900, RJ, Brazil

**Keywords:** thioureas, antiplatelet properties, *in silico* evaluation

## Abstract

The incidence of hematological disorders has increased steadily in Western countries despite the advances in drug development. The high expression of the multi-resistance protein 4 in patients with transitory aspirin resistance, points to the importance of finding new molecules, including those that are not affected by these proteins. In this work, we describe the synthesis and biological evaluation of a series of *N,N'*-disubstituted thioureas derivatives using *in vitro* and *in silico* approaches. New designed compounds inhibit the arachidonic acid pathway in human platelets. The most active thioureas (compounds **3d**, **3i**, **3m** and **3p**) displayed IC_50_ values ranging from 29 to 84 µM with direct influence over *in vitro* PGE_2_ and TXA_2_ formation. *In silico* evaluation of these compounds suggests that direct blockage of the tyrosyl-radical at the COX-1 active site is achieved by strong hydrophobic contacts as well as electrostatic interactions. A low toxicity profile of this series was observed through hemolytic, genotoxic and mutagenic assays. The most active thioureas were able to reduce both PGE_2_ and TXB_2_ production in human platelets, suggesting a direct inhibition of COX-1. These results reinforce their promising profile as lead antiplatelet agents for further *in vivo* experimental investigations.

## 1. Introduction

According to the World Health Organization, cardiovascular disease will be a leading cause of death in developed and developing countries by 2015. Currently, cardiovascular and thromboembolic events are already the major causes of death in Western countries [1].

Hemostasis is a widely studied topic due to the pathogenic nature of thrombotic and bleeding disorders [2,3]. The inappropriate activation of the hemostatic system contributes to the development of severe pathophysiological disorders, including the thromboembolic diseases, such as atherothrombosis and venous thromboembolism [4,5].

In general, antithrombotic drugs, including antiplatelet agents (e.g., clopidogrel, aspirin, tirofiban), are the primary treatment option for these diseases. However, they can lead to serious adverse reactions in some patients, including bleeding, neutropenia, thrombocytopenia and drug resistance [1,6,7,8,9,10]. Recent reports point to the highly expression of multidrug resistance protein 4 (MRP4) in platelets of patients with high incidence of transitory aspirin resistance [11]. MRP4 is an ATP-binding transporter which acts as an unidirectional pump for organic anionic compounds such as acetylsalicylate (the organic anion of aspirin) and is related to the extrusion of aspirin from platelets, reducing its pharmacological inhibition of cyclooxygenase-1 (COX-1) [12]. Suboptimal platelet inhibition by aspirin leads to an incomplete suppression of thromboxane generation, that is independently associated with increased risk of cardiovascular events [13].

Many non-steroidal anti-inflammatory drugs (NSAIDs) with antiplatelet activity due to COX-1 inhibition comprise the class of carboxylic acids, based on their ability to form strong salt-bridges with the guanidinium group of Arg120, located at the entrance of the cyclooxygenase site of COX-1 [14]. The role of this anchoring mechanism exemplifies that the ability of an acidic functional group to become anionic at physiological pH is a valuable structural and physicochemical feature of NSAIDs [15]. Unfortunately, the stable carboxylate anions formed by such compounds have a known tendency to interact with human organic anion transporters, such as multidrug resistance proteins (MRPs), that are often related to the undesired effects observed for many NSAIDs [16].

A variety of NSAIDs are able to interact with MRPs, with a higher specificity toward MRP4 [17], which suggests that other known NSAIDs bearing a carboxylic acid function are likely to be affected by biological resistance as observed for aspirin [11,12,13]. Therefore, based on the current literature, non-anionic COX-1 inhibitors may represent a potential alternative to current antiplatelet agents, prompting the development of a new drug class that has not been extensively investigated [18].

The thiourea moiety has been described as an important pharmacophore in a variety of promising chemical prototypes for drug development, including: anti-HIV (inhibitors of HIV capsid assembly) [19], anticancer [20], anticonvulsant [21], antimycobacterial [22], anti-HCV [23] and antimicrobial agents [24]. Recently the thiourea moiety was described for dual inhibition of both cyclooxygenase isoforms 1 and 2 with a 4-fold selectivity towards COX-2 active site [25], pointing its anti-inflammatory properties. The antithrombotic activity of this class of molecules was also explored as antagonists of the thrombin receptor PAR1 [26]. As the continued effort and identification of more effective compounds for the treatment of thrombotic diseases are still of considerable interest, in this work we developed a new series of non-anionic arylthiourea derivatives designed as novel antithrombotic agents targeting COX-1. Therefore, we synthesized a series of *N,N'*-disubstituted thioureas **3a**–**q** and analyzed their *in vitro* and *in silico* antithrombotic profiles. In addition we explored their potential molecular targets as well as their toxicological profile.

## 2. Results and Discussion

### 2.1. Chemistry

In order to develop thioureas having an unlikely propensity to act as substrates for MRPs, the carboxylate function was rejected for the final products and replaced by hydrophobic or H-bond acceptor (HBA) groups unable to produce organic anions at physiological pH. Therefore, we used aryl and alkyl groups substituents due to their hydrophobicity, pointed as a relevant feature to COX-1 selective inhibition when carboxylate groups are converted to less reactive acidic groups [18]. Co-substitutions of a potential lead molecule tailoring a HBA and a hydrophobic group was recently reported as an expressive approach for selective inhibition of cyclooxygenases, since it increases the molecular similarities to arachidonic acid by mimicking its 20-carbon hydrophobic ω-chain and the carboxylate group, which acts as a strong HBA group [25,27].

**Scheme 1:**
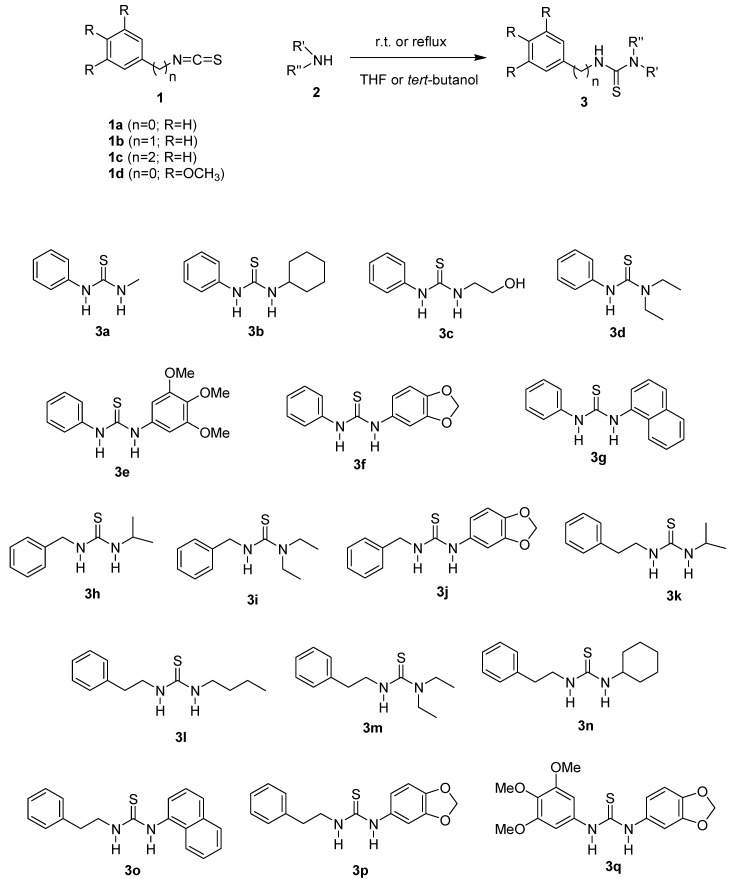
Synthesis of thiourea derivatives from isothiocyanates.

As a result of this rational design, a series of *N,N'*-disubstituted thioureas was obtained using the general procedure as shown in Scheme 1. The desired thioureas **3a**–**q** were readily prepared through the reaction of the appropriate isothiocyanate (**1a**–**d**) and an excess of amine **2**, in a THF or *tert*-butanol solution (Table 1). The products were obtained in good to excellent yields (82%–97%) and did not require any further purification after isolation from the crude reaction mixture by acidic extraction. The reactions used to obtain thioureas **3e**–**g**, **3j**, **3o**–**q**, between an aromatic amine and isothiocyanate required a reflux period due to the lower reactivity of the system, however these conditions did not compromise the yields (Table 1). The proposed structures of all prepared compounds were confirmed by FTIR, ^1^H-NMR, ^13^C-NMR and HRMS.

**Table 1:** Reaction conditions and yields for thioureas **3a**–**q** produced using Scheme 1.

Thiourea	Solvent	Temperature	Time (h)	Yield % ^a^
**3a**	THF	r.t.	4.0	97
**3b**	THF	r.t.	8.0	90
**3c**	THF	r.t.	6.0	89
**3d**	THF	r.t.	6.0	96
**3e**	*t*-BuOH	reflux	8.0	82
**3f**	*t*-BuOH	reflux	10.0	92
**3g**	*t*-BuOH	reflux	7.0	86
**3h**	THF	r.t.	5.0	85
**3i**	THF	r.t.	6.0	95
**3j**	*t*-BuOH	reflux	10.0	85
**3k**	THF	r.t.	5.0	89
**3l**	THF	r.t.	4.5	89
**3m**	THF	r.t.	4.0	88
**3n**	THF	r.t.	6.0	88
**3o**	*t*-BuOH	reflux	8.0	87
**3p**	*t*-BuOH	reflux	12.0	94
**3q**	*t*-BuOH	reflux	8.0	84

^a^ Isolated yield.

### 2.2. Biological Activity Evaluation

#### 2.2.1. Platelet Aggregation Assays

In physiological conditions, the release of arachidonic acid (AA) from the platelet membranes generates thromboxane A_2_ (TXA_2_), a potent platelet agonist, leading to shape-change, granule release and platelet aggregation [28,29]. The main enzymes responsible for the production of TXA_2_ in platelets are COX-1 and thromboxane synthase (TXS), both whose inhibition is known to prevent platelet aggregation [30,31].

**Figure 1:**
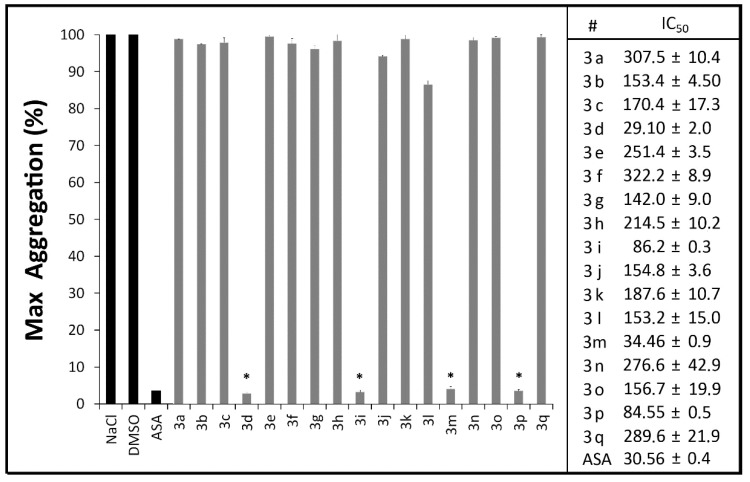
Antiplatelet profile of *N,N'*-disubstituted thiourea derivatives (100 μM) and aspirin (ASA) on *in vitro* platelet aggregation of human citrated platelet-rich plasma induced by arachidonic acid (AA). The results are the mean of two experiments performed in triplicate. * *p* < 0.05 (oneway ANOVA, Tukey test).

Aggregation of healthy human platelets induced by arachidonic acid revealed the antiplatelet activity of compounds **3d**, **3m**, **3i** and **3p**, that were able to inhibit AA-induced platelet aggregation in a range of 96%–98%, statistically similar to aspirin (97.5%) at the same concentration (100 μM) (Figure 1). These results pointed to the efficacy of our proposal on the production of novel antiplatelet thioureas.

The concentrations that caused 50% inhibition of platelet aggregation revealed compound **3d** (IC_50_ = 29.1 μM ± 2.0) as the most potent of the series followed by **3m** (IC_50_ = 34.5 μM ± 0.9), **3p** (IC_50_ = 84.6 μM ± 0.5) and **3i** (IC_50_ = 86.2 μM ± 0.3) (Figure 1). These data revealed the potency of these antiplatelet thiourea derivatives to inhibit the arachidonic acid pathway of platelet aggregation and suggests their ability to reduce the TXA_2_ production as a mechanism to impair normal platelet aggregation.

#### 2.2.2. Measurement of Plasma PGE_2_ and TXB_2_ Levels

The stable direct metabolite of PGE_2_, 13,14-dihydro-15-keto-Prostaglandin E_2_, is a known marker of the COX-1 activity [32,33]. By the measurement of total levels of this PGE_2_ metabolite in human platelet-rich plasma, we evaluated the feasibility of antiplatelet *N,N'*-disubstituted thioureas to inhibit COX-1 activity in healthy platelets (Table 2).

**Table 2:** Effects of novel antiplatelet *N,N'*-disubstituted thiourea derivatives (100 µM) on PGE_2_ and TXB_2_ production determined by Enzyme Immunoassay (EIA) in comparison to dimethylsulfoxide (DMSO, 1%), Ozagrel (100 µM), Aspirin (100 µM) and Indomethacin (100 µM).

#	Platelet Inhibition of PGE_2_ (%)	Platelet Inhibition of TXB_2_ (%)
**3d**	25.6 ± 5.9	74.2 ± 10.2
**3i**	21.1 ± 5.0	74.7 ± 5.5
**3m**	28.2 ± 5.9	91.2 ± 0.0
**3p**	7.9 ± 7.9	88.1 ± 5.5
Aspirin	47.5 ± 1.4	78.5 ± 5.0
Indomethacin	28.0 ± 1.6	99.8 ± 0.0
Ozagrel	-	98.0 ± 2.8
DMSO	0.0 ± 1.6	0.0 ± 0.0

Similar inhibition rates observed for indomethacin for PGE_2_ production were obtained by these antiplatelet thioureas at the same concentration (100 μM) suggesting a mechanism of action equally efficient to a known clinical drug. Aspirin (100 μM) showed higher inhibition of PGE_2_ production (47.5%) in comparison to indomethacin and the thiourea derivatives, which might be related to the non-competitive inhibition of COX-1 by aspirin through selective acetylation of Ser530 [34]. Previous studies have demonstrated the anti-inflammatory role of thiourea derivatives in xylene-induced ear swelling in mice, with mild COX-1 inhibition in comparison to aspirin [35], which suggested a mechanism of action that diverges from that of acetylsalicylic acid.

Thromboxane B_2_ (TXB_2_), a stable TXA_2_ metabolite, is widely used as a prognostic risk marker of platelet activation in cardiovascular disease, which is closely related to COX-1 and Thromboxane Synthetase activity [29,36]. Thus, the plasma levels of this metabolite were measured using an enzyme immunoassay (EIA) as described in the Experimental Section.

**Table 3:** Mutagenic and genotoxic activity of thiourea derivatives without metabolic activation evaluated by Ames test and SOS chromotest. The thiourea derivatives were dissolved in dimethylsulfoxide (DMSO) to perform assays. As positive control 4-NQO was used. Results of three different concentrations (10 µM, 100 µM and 500 µM).

Thiourea	Ames Test *S. typhimurium*				SOS Chromotest *E. coli*	
	TA97	TA98	TA100	TA102	PQ35	PQ37
**3a**	-	-	-	-	-	-
**3b**	-	-	-	-	-	-
**3c**	-	-	-	-	-	-
**3d**	-	-	-	-	-	-
**3e**	-	-	-	-	-	-
**3f**	-	-	-	-	-	-
**3g**	-	-	-	-	-	-
**3h**	-	-	-	-	-	-
**3i**	-	-	-	-	-	-
**3j**	-	-	-	-	-	-
**3k**	-	-	-	-	-	-
**3l**	-	-	-	-	-	-
**3m**	-	-	-	-	-	-
**3n**	-	-	-	-	-	-
**3o**	-	-	-	-	-	-
**3p**	-	-	-	-	-	-
**3q**	-	-	-	-	-	-
**4-NQO**	+	+	+	+	+	+
**DMSO**	-	-	-	-	-	-
**ASA**	-	-	-	-	-	-

The reduced production of TXB_2_ in platelet-rich plasma induced by the presence of the thiourea derivatives **3d**, **3i**, **3m** and **3p** presented similar inhibition rates in comparison to aspirin (78.5%), including the most active molecules **3m** (91%) and **3p** (88%) (Table 3). These data prompt the potential of this series of compounds as new non anionic antiplatelet agents against human plasma. Interestingly, we observed a direct correlation between the production of TXB_2_ and the length of the alkyl chain of the thiourea derivatives, whereby the phenethyl-substituted thiourea **3m** displayed a better inhibitory activity in comparison to its benzyl (**3i**) or phenyl (**3d**) analogs.

The ability of thioureas to reduce the platelet production of TXB_2_ and aggregation has been previously discussed [37], alongside with its vasoactive effects, such as reducing vascular hypertension at a similar magnitude to commercial non-steroidal anti-inflammatory drugs [38]. Studies suggest that the reduced TXB_2_ production by platelets after treatment with thiourea occurs due to the scavenging properties of hydroxyl radicals [39], a common property for thiourea compounds, which display antioxidant properties [40]. However, Scholz and collaborators suggested that COX-1 inhibition has no correlation to the inactivation of hydroxyl radicals but rather to structural properties of the ligands, able to improve binding affinity [41].

Currently, the literature reports that the basic structural requirements for selective inhibition of TXS are a 1-imidazolyl or a 3-pyridyl moiety at one end of the molecule (to block the heme iron through strong *pi*-cation interactions) [18,42] and a carboxylic acid at the other end to orient the molecule in a similar binding mode to prostaglandin H_2_ [43,44]. Importantly, both structural requirements are not fulfilled by any of the thiourea derivatives described herein, supporting the potential of this series as feasible COX-1 inhibitors.

**Figure 2:**
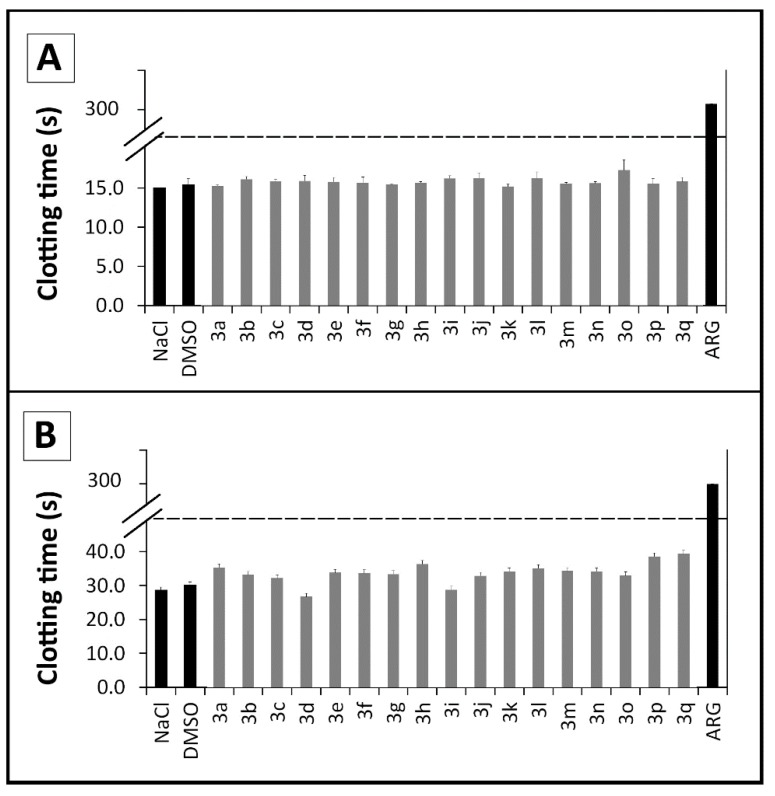
Evaluation of *N,N'*-disubstituted thiourea derivatives and argatroban (ARG) on the *in vitro* coagulation of human pooled plasma (*n* = 6) by activated partial thromboplastin time—aPTT (**A**) and prothrombin time—PT (**B**) assays. All derivatives and argatroban at 100 μM; DMSO at 1% and NaCl at 0.25 M.

#### 2.2.3. Coagulation Assays

To verify whether the plasmatic phase of blood coagulation was affected by these new compounds, we investigated the influence of these novel thiourea compounds **3a**–**q** in human pooled plasma (*n* = 6) on routine coagulation assays (activated partial thromboplastin time—aPTT, and prothrombin time—PT) [45]. Neither the extrinsic nor intrinsic pathways of the coagulation cascade were influenced by the presence of the compounds, suggesting that the antithrombotic profile of the compounds described herein relies on the direct impairment of platelet aggregation, thus differing from dual action molecules (Figure 2). This result reveals a small bleeding risk in comparison to dual acting molecules whose mechanism often lead to severe bleeding disorders as described in the literature [46,47].

#### 2.2.4. *In Vitro* Toxicity Assays

**Figure 3:**
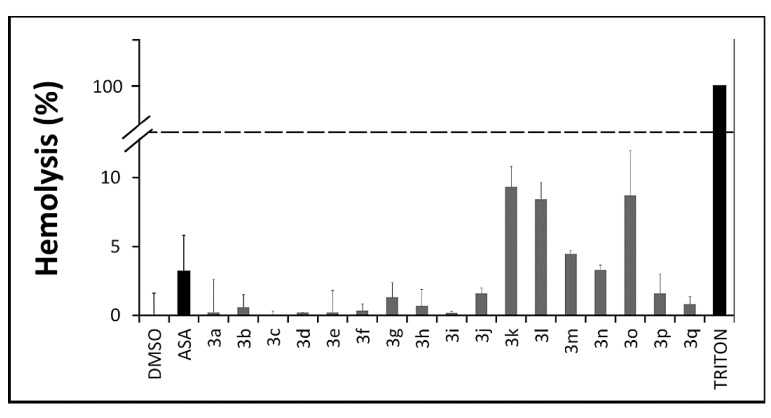
Evaluation of the hemolytic profile of *N,N'*-disubstituted thioureas through hemolysis assays over a 3 h period. Values below 10% are considered non hemolytic. All derivatives and ASA at 100 μM; DMSO and TRITON at 1%.

The literature shows that the interaction between chemical derivatives and erythrocytes may accelerate cell aging or lead to a mechanical premature destruction of these cellular integrity with release of hemoglobin. According to our erythrocyte lysis assay no significant hemolytic profile was observed for thioureas **3a**–**q** (Figure 3) after a 3 h incubation period (0%–9%). They were similar to aspirin (2%) (*p* ≤ 0.05) and not comparable to Triton X-100 (100%), used as a positive control. Fisher and collaborators [48] reported that hemolysis values below 10% are considered non-hemolytic, which frame these compounds within an acceptable toxicity profile [48,49,50].

This safe profile is reinforced by further toxicological studies which revealed no mutagenicity profiles against *Salmonella typhimurium* auxotroph mutant strains for all thiourea derivatives through reverse mutagenesis and histidine prototrophy (Ames test), compared to the positive control 4-nitro-quinoline 1-oxide (4NQO) [51,52], nor genotoxicity profiles, that was evaluated against *Escherichia coli* through the SOS chromotest [53]. This low risk profile is maintained even at the highest concentrations tested (500 μM, Table 3), revealing the safety profile of the series as promising for lead prototypes.

### 2.3. Computational Analysis

#### 2.3.1. Structure-Activity Relationship Studies (SAR)

In this work we evaluated different electronic parameters for all compounds (Table 4). Overall, the thiourea derivatives share a similar electronic profile for HOMO (−7.73 to −8.34 eV) and LUMO energies (2.18 to 3.72 eV). Interestingly, combining different hydrophobic groups with the HBA *N,N*-diethyl group, resulted in the most active molecules (compounds **3d**, **3i**, **3m** and **3p**) supporting our initial design strategy as an interesting method to develop antiplatelet agents bearing similarities to arachidonate [54].Co-substitutions of thioureas with the hydrophobic aliphatic chains at the side derived from isothiocyanate (Scheme 1) increase the molecular similarities to AA, by mimicking its 20-carbon hydrophobic ω-chain [27].

**Table 4:** The stereoelectronic properties of *N,N'*-disubstituted thioureas. DM = dipole moment (debye); PSA = polar surface area (Å^2^), clogP = octanol/water partition coefficient, MW = molecular weight, HBD = hydrogen bond donors, HBA = hydrogen bond acceptors.

#	E^HOMO^ (eV)	E^LUMO^ (eV)	DM	AREA (Å^2^)	VOLUME (Å^3^)	PSA	Lipinski Rule of Five			
							MW	clog P	HBD	HBA
**3a**	−8.34	3.12	5.82	196.34	172.56	20.237	166.25	1.99	2	3
**3b**	−8.27	3.15	5.78	272.63	251.41	18.336	234.37	3.52	2	3
**3c**	−8.24	3.22	5.66	225.92	198.74	39.126	196.27	1.48	3	4
**3d**	−8.19	3.01	5.21	248.09	227.35	11.734	208.33	3.05	1	3
**3e**	−7.95	2.7	6.94	331.01	315.91	40.151	318.4	3.28	2	6
**3f**	−7.73	2.85	6.16	272.97	259.97	37.832	272.33	3.44	2	5
**3g**	−7.64	2.13	6.43	293.95	285.88	20.889	278.38	4.66	2	3
**3h**	−8.26	3.52	6.17	257.56	228.28	19.745	208.33	2.72	2	3
**3i**	−8.19	3.58	5.60	276.39	247.98	9.5490	222.36	3.12	1	3
**3j**	−8.10	3.16	5.94	303.20	281.49	35.421	286.36	3.51	2	5
**3k**	−8.26	3.65	5.78	276.49	246.67	19.462	222.36	3.00	2	3
**3l**	−8.27	3.64	5.62	295.98	265.13	20.208	236.38	3.58	2	3
**3m**	−8.21	3.72	5.15	294.52	266.42	9.4050	236.38	3.40	1	3
**3n**	−8.21	3.68	5.79	312.13	288.54	18.806	262.42	3.89	2	3
**3o**	−7.92	2.18	5.66	343.86	325.92	18.886	306.43	5.00	2	3
**3p**	−8.05	3.15	5.82	320.69	299.61	34.234	300.38	3.79	2	5
**3q**	−7.85	2.69	6.86	356.02	341.05	56.282	362.41	3.06	2	8

Substitutions on the thiourea moiety by replacing acceptor groups at R' and R'' lead to less active compounds (**3a**, **3b**, **3g**, **3h**, **3k**, **3l**, **3n** and **3o**), suggesting that the prevalence of a hydrogen-bond acceptor group in R” over of a donor group is favorable to the antiplatelet activity of this series. In addition, compounds with a hydrogen-bond acceptor group (R' and R'') in the absence of aliphatic chain (**3e**, **3f**) lack antiplatelet activity as well as compounds bearing HBA groups at the both sides of thiourea moiety (**3q**), which reinforce the importance of having a design resulted from the combination of a HBA group with a hydrophobic group. Increasing the length of the aliphatic chain of methylenedioxy-substituted thiourea, from phenyl (**3f**) and benzyl (**3j**) to phenethyl (**3p**) led to an improvement of antiplatelet activity (IC_50_ = 84.5 μM—Figure 1).The increase of chain length of bioactive molecules for improved lipophilicity have been reported as a relevant parameter to inhibit human platelet aggregation [55], as it increases the affinity of the molecule towards COX-1 and facilitates passive transport across biological membranes [15]. Interestingly, substitution of the methyl group in thiourea **3a** by an *N,N*-diethyl group (**3d**) replaced the H-bond donor profile by an acceptor one, leading to a successful combination that resulted in the most active compound (Table 4). Increasing chain length from phenyl (**3d**) to benzyl-substituted thiourea (**3i**) reduced potency by nearly three-fold, while no expressive reduction was observed for the phenethyl-substituted thiourea (**3m**) (Figure 1).

The evaluation of this novel series of thiourea derivatives reinforced lipophilicity as an important feature for modulating the antiplatelet activity, since the substitution by longer hydrophobic groups at the region derived from the isothiocyanate, preferably opposed by a polar electron-rich hydrogen-bond acceptor group (e.g., methylenedioxy or *N,N*-diethyl groups at R' and R''), increased the biological activity of all compounds, except for thiourea **3d**, which may have a different mechanism of action apart from the establishment of low ∆G complexes with the enzyme. Our data suggests the importance of the substitution of the thiourea moiety with a *N,N*-diethyl group, leading to a high antiplatelet activity profile.

Literature describes that COX-1 is located into dense tubule structures inside platelets [56]. Primary eicosanoids, such as arachidonate and thromboxane A_2_, are not able to diffuse passively through platelets membranes and require active transport by human organic anion transporters such as MRP4 [57]. This suggests the importance of cell permeability for non-anionic compounds such as the thioureas presented herein. Recent studies also point to the relevance of hydrophobicity management for COX-1 selective inhibition when carboxylate groups of known ligands are converted to less reactive groups [18] and our data supports the accuracy of that point.

#### 2.3.2. Docking Analysis

**Figure 4:**
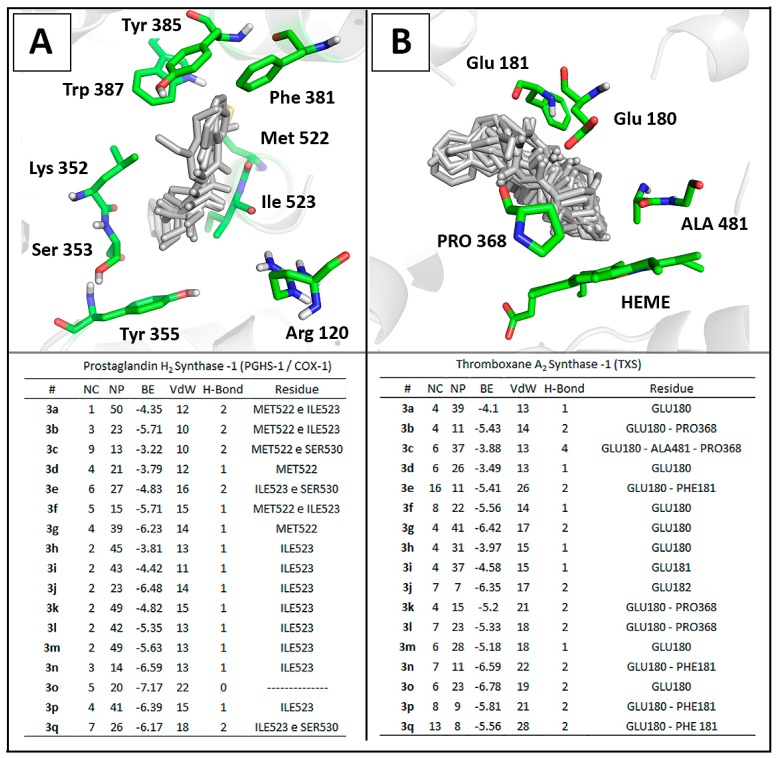
Comparison of molecular docking complexes of *N,N'*-disubstituted thioureas with (**A**) the crystallographic structure of ovine COX-1 (PDB ID: 2OYE) and (**B**) a three-dimensional model of human thromboxane synthase [29]. Interaction panels include number of clusters (NC), number of poses in the lowest energy cluster (NP), binding energy score (BE) and intermolecular interactions: Van der Waals (VdW) and hydrogen bonds (H-bond).

All *N,N'*-disubstituted thiourea derivatives were docked into the active site of the ovine COX-1 (PDB ID: 2OYE) and Thromboxane Synthase (TXS) [44]. The theoretical complexes were evaluated and ranked by number of clusters, number of conformations at the lowest energy cluster, binding energy, and number of interactions (Figure 4). Notably, all compounds presented a lower number of different clusters in COX-1 when compared to the TXS derivatives complexes which lead to an increased numbers of stable poses observed for COX-1. This data corroborate to previous studies that determine the structural determinants for TXS inhibition [43] which are not addressed by any of the thiourea derivatives described herein. This theoretical data reinforce the feasibility of these molecules as COX-1 inhibitors.

Docking results showed that phenethyl-substituted molecules **3k**–**p** formed complexes with higher stability to the enzyme in comparison to their phenyl or benzyl-substituted analogs (Figure 4). This results reinforced the correlation between lipophilicity and binding affinity to COX-1 [18]. Interestingly, the carbonyl backbone of Ile523 from COX-1 was able to establish hydrogen bonds (2.0 Å) with NH group from the thiourea moiety in 13 of the 17 thiourea complexes, having **3c**, **3d**, **3g** and **3o** as exceptions (Figure 4).

This feature might be involved in the recognition and activity of thiourea derivatives against COX-1. Literature points that the I523V mutation in COX-1 decreases the cyclooxygenase activity by 30% suggesting the catalytic relevance of Ile523 in COX-1 to the conversion of AA into PGG_2_ [58]. In COX-1, Ile523 reduces the space of the hydrophobic cavity that is available in COX-2 due to the replacement of isoleucine by valine [59]. Furse *et al*. described the mechanism using a molecular dynamics approach and reported that the arachidonic acid (AA) is oxygenated by PGHS1, revealing that AA displays considerably less conformational flexibility in COX-1 in comparison to COX-2. This points the importance of Ile523 to maintain AA in a catalytically favorable orientation towards Tyr385 during the enzymatic process [60]. This process might be impaired by stable thiourea complexes formed.

The enzyme-ligand complex analysis revealed that phenyl, benzyl and phenethyl groups were orientated towards the hydrophobic channel near Tyr385 for all compounds, with the other substituent at R' and R'', such as 3,4-methylenedioxyphenyl or *N,N-diethyl*, orientated towards the active site entrance to meet Arg120 and Tyr355 (Figure 4). This orientation is similar to those described for many NSAIDs. One of the most important interaction involves Arg120 in a salt-bridge interaction with the ligand carboxylate anion and lipophilic groups buried into the hydrophobic channel of the enzyme [61,62,63].

**Figure 5:**
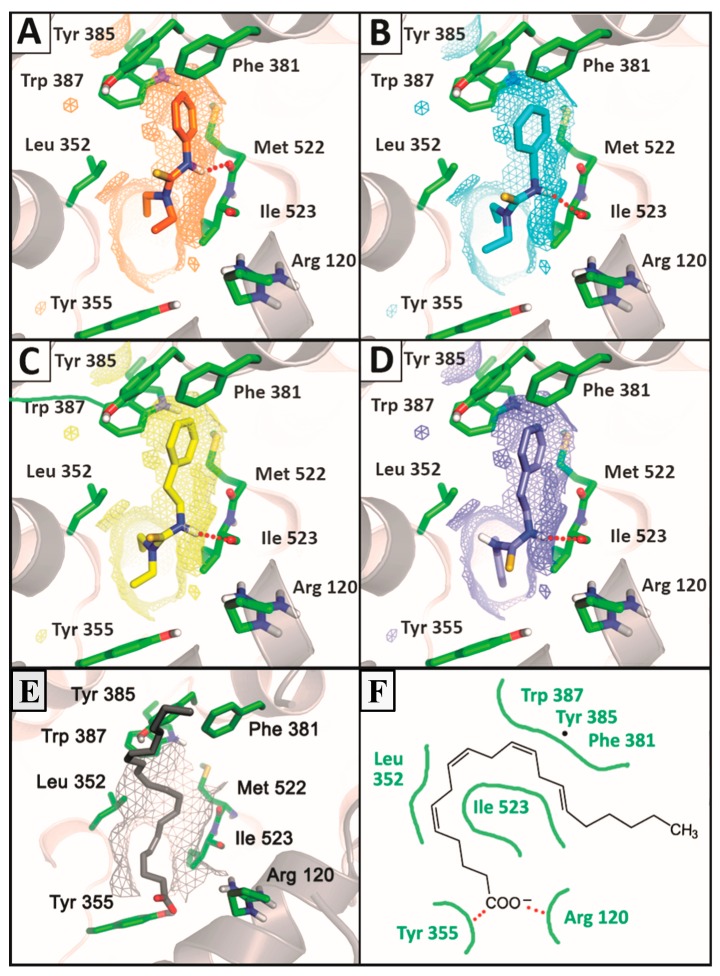
Comparison of the most stable poses observed for the active compounds **3d** (**A**), **3i** (**B**), **3m** (**C**), **3p** (**D**) and arachidonic acid (**E**,**F**) with the crystallographic structure of ovine COX-1 (PDB ID: 2OYE). Hydrogen bounds are shown as red balls.

Such a feature has direct relationship to the biological activity of these agents accordingly to crystal structures from ovine COX-1 complexed with AA. The carboxylate group of the substrate interacts with the guanidinium group of Arg120 and establishes a hydrogen bond to Tyr355, while the aliphatic backbone go toward the hydrophobic channel of the catalytic site, bending in the vicinity of Tyr385 [64]. Such binding mode is achieved preferably by phenethyl-substituted compounds in addition to a co-substitution by methylenedioxy (**3p**) or *N,N*-diethyl groups (**3m**) and generates high stable complexes with COX-1. This suggests a feasible blockage of active site from arachidonic acid recognition (Figure 5).

The phenyl-substituted thiourea **3d** displayed the best antiplatelet profile of the series with the lowest influence on the production of TXB_2_ by activated platelets. Docking analysis revealed that this compound binds to the catalytic site of COX-1 with lower affinity in comparison to benzyl (**3i**) and phenethyl-substituted (**3m**) derivatives (Figure 5). However, due to the hydrophobicity of the phenyl group and the hydrogen bond acceptor profile of *N,N*-diethyl group, thiourea **3d** is able to display a similar orientation to arachidonic acid and the phenethyl-substituted derivatives in the active site of the enzyme (Figure 5). In addition, the smaller size of the phenyl group allows the thiourea moiety to be inserted closer to Tyr385 in comparison to benzyl or phenethyl-substituted molecules, whereas it is able to establish a H-bond with Met522 rather than Ile523.

COX-1 inhibitors are known to bind near the top of the hydrophobic channel and affect interactions with Tyr385 [65]. According to literature, the COX-1-mediated catalysis of arachidonic acid requires the formation of a tyrosyl radical from Tyr385 through oxidation of the heme iron after reaction with endogenous peroxides [66]. The tyrosyl radicals are transient and rapidly dissipated by reductants, suggesting that COX-1-mediated catalysis might be vulnerable to suppression by antioxidant molecules [40,67]. Wu *et al*., reported that the carboxylic acid of diclofenac binds in a non-canonical way to the top of the hydrophobic channel, in an arrangement that place strong charges next to the tyrosyl radical, reducing its radical lifetime. Differently, flurbiprofen establish a canonical salt bridge with Arg120, with the hydrophobic phenyl ring near the hydroxyl group with low radical perturbation but high binding affinity [67]. Thus, the inhibition of COX-1 can be achieved by high-affinity protein-ligand complexes, as observed by **3i**, **3m** and **3p**, in the canonical way [67].

**Figure 6:**
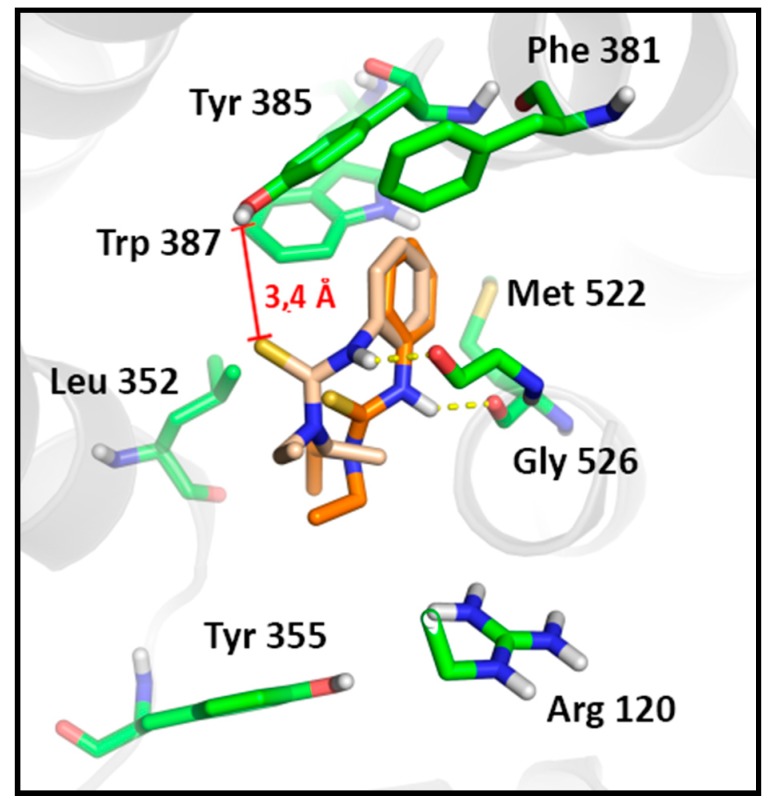
Comparison of the different stable poses observed for the antiplatelet *N,N'*-disubstituted thiourea derivative (**3d**) on COX-1. The most stable pose of the first cluster (Orange, Binding Energy = −3.79) in comparison to the most stable pose of the second cluster (Pink, Binding Energy = −3.52). Hydrogen bounds are shown as yellow dashes.

To confirm the hypothesis that thiourea **3d** is able to perturb the dynamics of the tyrosyl radical formation, we evaluated other stable poses of this compound in the active site of COX-1, based on its lower stability in comparison to other active molecules. The second most stable cluster of thiourea **3d** in COX-1 presented the same number of conformations as the most stable one, but with a lower affinity (Binding energy = −3.52 kcal/mol). Such binding mode reveals greater proximity of the thiourea moiety towards Tyr385 hydroxyl group (from 3.7 to 3.4 Å) in a range of poses that are stabilized by a 2.1 Å hydrogen bound with Gly526, while maintaining the hydrophobic contacts of the phenyl ring (Figure 6). This data corroborates the antiplatelet profile of compound **3d** and also confirm the hypothesis that the activity profile of **3d** arises from its modulatory effect onTyr385. In addition these results corroborate with the Wu *et al*. studies, revealing the feasibility of a different mechanism of action on COX-1 for molecules that are able to display a non-canonical binding mode to COX-1 [67]. These data reinforce the relevance of novel *N,N'*-disubstituted thiourea derivatives as non-anionic antiplatelet agents.

Several non-anionic COX-1 inhibitors were recently described with IC_50_ values ranging from micromolar concentrations (26 µM—rofecoxib, 15 µM—celecoxib, 0.8 µM—TFAP) to nanomolar concentrations as biphenyl analogues of sulindac (570 nM) [18]. However even some of those high-selectivity inhibitors may have off-target effects, as described by sulindac derivatives. The series of thiourea derivatives described herein presents new active antiplatelet compounds with IC_50_ values at a micromolar ranging from 29 to 84 µM, with no traces of hemolytic or genotoxic activity and no crosslink activity at the plasmatic coagulation cascade. Therefore, we reinforce the potential of the thiourea moiety as a promising pharmacophore for the design of high-selective and safe NSAIDs.

## 3. Experimental Section

### 3.1. General Information

All chemicals were obtained from commercial suppliers and were used without further purification. ^1^H- and ^13^C-NMR spectra were recorded on an Avance 200 MHz spectrometer (Bruker, Billerica, MA, USA) using CDCl_3_ or DMSO-*d_6_* as the solvent. Standard Bruker software was used throughout. Chemical shifts were given in ppm (δ scale) and coupling constants (*J*) were given in hertz (Hz). The IR spectra were obtained on a IRPrestige-21 FTIR spectrometer (Shimadzu, Tokyo, Japan) using KBr pellets. High-resolution mass spectra (HRMS) were obtained on a Bruker microTOF II mass spectrometer using ESI. Melting points were recorded on a Shimadzu DSC-60 thermal analyzer at a heating rate of 10 °C/min, room temperature to 200 °C, under a nitrogen flow rate of 50 mL/min and using an aluminum standard. Analytical TLC (silica gel, aluminum sheets 60 F254, Merck, Darmstadt, Germany) was performed using ethyl acetate/hexane (1:5 v/v) as the eluent.

#### General Procedure for the Preparation of Thioureas **3a**–**q**

To a solution of isothiocyanate **1** (1.0 mmol) in THF or *tert*-butanol (10 mL) was added the appropriate amine **2** (1.2 mmol). The mixture was stirred at room temperature or refluxed. After the reaction was completed (TLC), the solvent was evaporated and CH_2_Cl_2_ (10 mL) was added. The organic phase was washed with 5% HCl_(aq)_ (3 × 10 mL), dried with anhydrous Na_2_SO_4_ and evaporated to dryness to afford the pure product **3**, which did not require any purification.

*N-Methyl-N'-phenylthiourea* (**3a**) [68]. White solid; mp 115–117 °C (Lit. [68] 110–112 °C); IR (KBr): 3263, 3161, 2989, 1595, 1541, 1519, 1255, 1033, 725, 690 cm^−1^; ^1^H-NMR (CDCl_3_) δ 8.28 (br s, 1H), 7.47–7.38 (m, 2H), 7.34–7.21 (m, 3H), 6.12 (br s, 1H), 3.11 (d, *J* = 4.7 Hz, 3H); ^13^C-NMR (CDCl_3_) δ181.7, 136.4, 130.3, 127.4, 125.5, 32.2; HRMS-ESI: *m/z* [M+Na]^+^ calculated for C_8_H_10_N_2_S: 189.0457; found: 189.0465.

*N-Cyclohexyl-N'-phenylthiourea* (**3b**) [69]. White solid; mp 149–151 °C (Lit. [69] 144–145 °C); IR (KBr): 3269, 3240, 2937, 2852, 1541, 1508, 1450, 1147, 983 cm^−1^; ^1^H-NMR (CDCl_3_) δ 8.12 (br s, 1H), 7.43 (t, *J* = 7.6 Hz, 2H), 7.32–7.18 (m, 3H), 5.94 (br d, 1H), 4.39–4.14 (m, 1H), 2.14–2.02 (m, 2H), 1.79–1.63 (m, 3H), 1.49–1.30 (m, 2H), 1.24–1.03 (m, 3H); ^13^C-NMR (50 MHz, CDCl_3_) δ 179.3, 136.4, 130.4, 127.2, 125.1, 54.1, 32.7, 25.6, 24.8; HRMS-ESI: *m/z* [M+Na]^+^ calculated for C_13_H_18_N_2_S: 257.1083; found: 257.1088.

*N-(2-Hydroxyethyl)-N'-phenylthiourea* (**3c**) [70]. White solid; mp 139–141 °C; IR (KBr): 3361, 3184, 3001, 2949, 1544, 1253, 1056, 929, 690 cm^−1^; ^1^H-NMR (DMSO-*d_6_*) δ 9.60 (br s, 1H), 7.70 (br s, 1H), 7.47–7.42 (m, 2H), 7.35–7.27 (m, 2H), 7.14–7.05 (m, 1H), 4.82 (br s, 1H), 3.56–3.54 (m, 4H); ^13^C-NMR (50 MHz, DMSO-*d_6_*) δ 180.5, 139.3, 128.5, 124.0, 123.0, 59.3, 46.4; HRMS-ESI: *m/z* [M+Na]^+^ calculated for C_9_H_12_N_2_OS: 219.0562; found: 219.0563.

*N-N-Diethyl-N'-phenylthiourea* (**3d**). Yellow oil; IR (film): 3259, 2975, 2931, 2084, 1596, 1525, 1376, 1350, 1137, 1095, 761, 698 cm^−1^, ^1^H-NMR (CDCl_3_) δ7.35–7.24 (m, 4H), 7.20–7.11 (m, 1H), 7.02 (br s, 1H), 3.69 (q, *J* = 7.1 Hz, 4H), 1.25 (t, *J* = 7.1 Hz, 6H); ^13^C-NMR (CDCl_3_) δ 180.9, 139.9, 128.7, 125.9, 125.8, 45.8, 12.8; HRMS-ESI: *m/z* [M+Na]^+^ calculated for C_11_H_16_N_2_S: 231.0926; found: 231.0932.

*N-Phenyl-N'-(3,4,5-trimethoxyphenyl)thiourea* (**3e**). White solid; mp 164–166 °C; IR (KBr): 3278, 3159, 2999, 2941, 1597, 1537, 1506, 1255, 1134, 997, 696 cm^−1^; ^1^H-NMR (CDCl_3_) δ 8.05 (br s, 2H), 7.42–7.37 (m, 4H), 7.34–7.20 (m, 1H), 6.63 (s, 2H), 3.84 (s, 9H); ^13^C-NMR (CDCl_3_) δ 180.0, 153.9, 137.4, 137.2, 132.7, 129.6, 127.1, 125.3, 103.3, 61.1, 56.5; HRMS-ESI: *m/z*[M+Na]^+^ calculated for C_16_H_18_N_2_O_3_S: 341.0930; found: 341.0940.

*N-3,4-Methylenedioxyphenyl-N'-phenylthiourea* (**3f**). Dark solid; mp 140–142 °C; IR (KBr): 3325, 3161, 2957, 1547, 1501, 1485, 1373, 1034, 926, 743, 694 cm^−1^; ^1^H-NMR (CDCl_3_) δ 8.18–7.83 (m, 2H), 7.56–7.17 (m, 5H), 6.99–6.65 (m, 3H), 5.98 (s, 2H); ^13^C-NMR (CDCl_3_) δ 180.6, 148.5, 147.1, 137.4, 130.8, 129.6, 127.1, 125.4, 119.7, 108.7, 107.8, 102.0; HRMS-ESI: *m/z* [M+Na]^+^ calculated for C_14_H_12_N_2_O_2_S: 295.0512; found: 295.0520.

*N-α-Naphthyl-N'-phenylthiourea* (**3g**) [71]. White solid; mp 155–157 °C (Lit. [71] 162–163 °C); IR (KBr): 3339, 3134, 2965, 1593, 1537, 1506, 1395, 1275, 1221, 775, 696, 646 cm^−1^; ^1^H-NMR (DMSO-*d_6_*) δ 9.96 (br s, 2H), 8.06–7.91 (m, 2H), 7.88–7.82 (m, 1H), 7.65–7.48 (m, 6H), 7.40–7.29 (m, 2H), 7.16–7.08 (m, 1H); ^13^C-NMR (DMSO-*d_6_*) δ 181.3, 139.7, 135.2, 133.9, 129.9, 128.4, 128.3, 128.1, 126.1, 126.0, 125.6, 124.4, 123.9, 123.6, 123.1; HRMS-ESI: *m/z* [M+Na]^+^ calculated for C_17_H_14_N_2_S: 301.0770; found: 301.0775.

*N-Benzyl-N'-isopropylthiourea* (**3h**) [72]. Pale yellow solid; mp 121–123 °C (Lit. [72], 122–123 °C); IR (KBr): 3363, 3145, 2974, 1539, 1298, 972, 744, 640 cm^−1^; ^1^H-NMR (CDCl_3_) δ 7.51–7.24 (m, 5H), 6.31 (br s, 1H), 5.91 (br s, 1H), 4.60 (d, *J* = 5.2 Hz, 2H), 4.29–4.07 (m, 1H), 1.14 (d, *J* = 6.5 Hz, 6H); ^13^C-NMR (CDCl_3_) δ 180.9, 137.2, 129.0, 128.0, 127.7, 48.5, 46.4, 22.6; HRMS-ESI: *m/z* [M+Na]^+^ calculated for C_11_H_16_N_2_S: 231.0926; found: 231.0926.

*N-Benzyl-N',N'-diethylthiourea* (**3i**) [73]. Yellowoil; IR (film): 3312, 2975, 2930, 1532, 1441, 1347, 1280, 1136, 862, 698 cm^−1^; ^1^H-NMR (CDCl_3_) δ 7.46–7.22 (m, 5H), 5.55 (br s, 1H), 4.87 (d, *J* = 4.9 Hz, 2H), 3.66 (q, *J* = 7.1 Hz, 4H), 1.23 (t, *J* = 7.1 Hz, 6H); ^13^C-NMR (CDCl_3_) δ 180.6, 138.4, 128.9, 128.0, 127.7, 50.3, 45.3, 12.8; HRMS-ESI: *m/z* [M+Na]^+^ calculated for C_12_H_18_N_2_S: 245.1083; found: 245.1077.

*N-Benzyl-N'-(3,4-methylenedioxyphenyl)thiourea* (**3j**). Dark solid; mp 114–116 °C; IR (KBr): 3317, 3161, 3005, 1546, 1523, 1487, 1246, 1197, 1039, 923, 700 cm^−1^; ^1^H-NMR (CDCl_3_) δ 8.06 (br s, 1H), 7.54–7.18 (m, 5H), 6.91–6.63 (m, 3H), 6.23 (br s, 1H), 5.97 (s, 2H), 4.84 (d, *J* = 5.4 Hz, 2H); ^13^C-NMR (CDCl_3_) δ 181.6, 148.9, 147.3, 137.6, 129.7, 128.9, 127.8, 127.7, 119.9, 109.1, 107.6, 102.0, 49.5; HRMS-ESI: *m/z* [M+Na]^+^ calculated for C_15_H_14_N_2_O_2_S: 309.0668; found: 309.0667.

*N-Isopropyl-N'-(2-phenethyl)thiourea* (**3k**). White solid; mp 126–128 °C; IR (KBr): 3298, 3234, 2971, 2922, 2360, 2333, 1564, 1519, 1183, 1003, 773, 694 cm^−1^; ^1^H-NMR (CDCl_3_) δ 7.37–7.15 (m, 5H), 5.79 (br s, 2H), 4.12–3.87 (m, 1H), 3.71 (q, *J* = 6.6 Hz, 2H), 2.92 (t, *J* = 6.6 Hz,2H), 1.15 (d, *J* = 6.4 Hz, 6H); ^13^C-NMR (CDCl_3_) δ 180.7, 138.6, 129.0, 128.9, 126.9, 46.0, 45.8, 35.4, 22.6; HRMS-ESI: *m/z* [M+Na]^+^ calculated for C_12_H_18_N_2_S: 245.1083; found: 245.1082.

*N-Butyl-N'-(2-phenethyl)thiourea* (**3l**). Pale yellow solid; mp 61–63 °C; IR (KBr): 3253, 2958, 2933, 2361, 1568, 1434, 1408, 1246, 1186, 1094, 1003, 977 cm^−1^; ^1^H-NMR (CDCl_3_) δ 7.37–7.20 (m, 5H), 5.94 (br s, 1H), 5.79 (br s,1H), 3.77 (q, *J* = 6.5 Hz, 2H), 3.24–3.22 (m,2H), 2.92 (t, *J* = 6.5, 2H), 1.59–1.42 (m, 2H), 1.38–1.23 (m, 2H), 0.90 (t, *J* = 7.1 Hz, 3H); ^13^C-NMR (CDCl_3_) δ 181.7, 138.6, 128.9 (2C), 126.9, 45.9, 43.9, 35.4, 31.0, 20.2, 13.8; HRMS-ESI: *m/z* [M+Na]^+^ calculated for C_13_H_20_N_2_S: 259.1239; found: 259.1242.

*N,N-diethyl-N'-(2-phenethyl)thiourea* (**3m**) [74]. White solid; mp 72–74 °C (Lit. [74] 67–69 °C); IR (KBr): 3393, 2930, 2360, 2332, 1541, 1453, 1410, 1330, 1269, 1135, 756 cm^−1^; ^1^H-NMR (CDCl_3_) δ 7.35–7.20 (m, 5H), 5.33 (br s, 1H), 3.90 (q, *J* = 6.5 Hz, 2H), 3.54 (q, *J* = 7.1 Hz, 4H), 2.96 (t, *J* = 6.5 Hz, 2H), 1.09 (t, *J* = 7.1 Hz, 6H); ^13^C-NMR (CDCl_3_) δ 180.3, 139.1, 128.9, 128.8, 126.7, 46.8, 45.1, 35.2, 12.6; HRMS-ESI: *m/z* [M+Na]^+^ calculated for C_13_H_20_N_2_S: 259.1239; found: 259.1232.

*N-Cyclohexyl-N'-(2-phenethyl)thiourea* (**3n**). Pale yellow solid; mp 109–111 °C; IR (KBr): 3289, 3224, 3047, 2930, 2856, 1547, 1319, 1193, 1123, 1058, 713 cm^−1^; ^1^H-NMR (CDCl_3_) δ 7.36–7.12 (m, 5H), 5.89 (br s, 2H), 3.77–3.68 (m, 3H), 2.91 (t, *J* = 7.0 Hz, 2H), 1.98–1.57 (m,5H), 1.44–0.98 (m, 5H); ^13^C-NMR (CDCl_3_) δ 180.4, 138.6, 128.9 (2C), 126.8, 52.9, 45.7, 35.3, 32.8, 25.4, 24.8; HRMS-ESI: *m/z* [M+Na]^+^ calculated for C_15_H_22_N_2_S: 285.1396; found: 285.1396.

*N-(α-Naphthyl)-N'-(2-phenethyl)thiourea* (**3o**). White solid;mp 155–157 °C; IR (KBr): 3364, 3314, 3309, 2362, 1596, 1537, 1495, 1342, 1266, 1013, 772, 696 cm^−1^; ^1^H-NMR (CDCl_3_) δ8.34 (br s, 1H), 8.05–7.80 (m, 3H), 7.60–7.46 (m, 2H), 7.43–7.35 (m, 1H), 7.25–7.19 (m, 1H), 7.11–7.00 (m,3H), 6.93–6.86 (m, 2H), 5.66 (br s, 1H), 3.77 (q, *J* = 6.6 Hz, 2H), 2.75 (t, *J* = 6.6 Hz, 2H); ^13^C-NMR (CDCl_3_) δ181.4, 138.2, 134.6, 131.5, 130.0, 128.8, 128.5, 128.4, 128.3, 127.4, 127.0, 126.4, 125.6, 125.2, 122.5, 46.2, 34.8; HRMS-ESI: *m/z* [M+Na]^+^ calculated for C_19_H_18_N_2_S: 329.1083; found: 329.1074.

*N-(3,4-Methylenedioxyphenyl)-N'-(2-phenethyl)thiourea* (**3p**). Dark solid; mp 126–128 °C; IR (KBr): 3305, 3196, 3026, 1548, 1535, 1500, 1487, 1244, 1037, 931, 858 cm^−1^; ^1^H-NMR (CDCl_3_) δ 7.89 (br s, 1H), 7.42–7.07 (m, 5H), 6.69 (d, *J* = 7.9 Hz, 1H), 6.52–6.47 (m, 2H), 5.99 (s, 2H), 5.87 (br s, 1H), 3.84 (q, *J* = 6.6 Hz, 2H), 2.90 (t, *J* = 6.6 Hz, 2H); ^13^C-NMR (CDCl_3_) δ 181.1, 148.8, 147.2, 138.6, 129.5, 128.9, 128.8, 126.8, 119.8, 109.0, 107.5, 102.0, 46.3, 35.0; HRMS-ESI: *m/z* [M+Na]^+^ calculated for C_16_H_16_N_2_O_2_S: 323.0825; found: 323.0832.

*N-(3,4-Methylenedioxyphenyl)-N'-(3,4,5-trimethoxyphenyl)thiourea* (**3q**). Dark solid; mp 142–144 °C; IR (KBr): 3313, 3196, 2999, 2835, 1600, 1541, 1236, 1124, 1033, 995 cm^−1^; ^1^H-NMR (CDCl_3_) δ 8.04 (br s, 2H), 6.93–6.92 (m, 1H), 6.79–6.77 (m, 2H), 6.63 (s, 2H), 5.99 (s, 2H), 3.83–3.82 (m, 9H); ^13^C-NMR (CDCl_3_) δ 180.4, 153.8, 148.4, 146.9, 137.0, 132.9, 131.0, 119.5, 108.6, 107.7, 103.2, 101.9, 61.0, 56.4; HRMS-ESI: *m/z* [M+Na]^+^ calculated for C_17_H_18_N_2_O_5_S: 385.0829; found: 385.0826.

### 3.2. Pharmacology

#### 3.2.1. Platelet Aggregation Assays

The human blood samples were obtained from adult volunteers, who abstained from the use of drugs or other substances that could interfere with the experiment for at least 15 days prior. Local ethical committee approved the procedure under protocol 140/10 and 177/11. The platelet rich (PRP) and poor (PPP) plasma were prepared by differential centrifugation and platelet aggregation was monitored using the turbidimetric method described by Born and Cross with an aggregometer 4-Pack^®^ (Helena Laboratories, Beaumount, TX, USA) [75]. Different concentrations of the synthetic derivatives and the vehicle (DMSO 1%) were pre-incubated for 2 min before addition of the agonists (500 μM arachidonic acid and 5 μg/mL collagen). The platelet aggregation tests were performed in triplicate and the data were statistically analyzed [76,77]. The concentration capable of inhibiting 50% of platelet aggregation was obtained through non-linear regression of the dose-response curve obtained for each compound (0, 10, 50 and 100 µM) performed in Matlab version 2014 (Mathworks^©^, Natick, MA, USA) with R^2^ = 0.99.

#### 3.2.2. Clotting Assay

For tests of activated partial thromboplastin time (aPTT) and Prothrombin Time (PT) donor citrated plasma samples were obtained from Antônio Pedro University Hospital (pool = 6 donors). No disturbances to the hemostatic system were found, and the international normalized ratio (INR was expressed for coagulation less than or equal to 1.3. Assays were performed using the coagulation analyzer CoagLab^®^IV (Beijing Shining Sun Technology Co., Ltd., Beijing, China) as described by described by Sathler *et al*. [44].

#### 3.2.3. Measurement of Plasma TXB_2_ Levels

Plasma samples containing 100 μM of each thiourea with antiplatelet activity were used to determine the concentrations of thromboxane B_2_, an index of *in vitro* COX-1 activity [78]. Briefly, 10 μL of arachidonic acid (500 μM) were added to platelet-rich plasma samples containing each active derivative (100 μM) under stir conditions. After five minutes the suspensions were mixed with 3 μL Indomethacin (10 mM) stop-solution. By sonication for 15 min, platelets were disrupted, and the resulting homogenates were centrifuged at 2000 *g* for 10 min to allow complete release of TXB2. Plasma levels of TXB_2_ were measured in duplicate by competitive immunoassay using commercially available kits from EIA Cayman (TXB_2_ EIA Kit, Cayman Chemical Co, Ann Arbor, MI, USA) according to the manufacturer’s instructions. A threshold of 25.75 ng/mL was defined as control plasma levels of TXB_2_ after platelet disrupture.

#### 3.2.4. Measurement of Plasma PGE_2_ Levels

Plasma samples containing 100 μM of each thiourea with antiplatelet activity were used to determine the concentrations of prostaglandin H_2_, an index of *in vitro* COX-1 activity [78]. Briefly, ozagrel (3 μL, 100 μM) were added to stop TXS activity and the aggregation were triggered with 10 μL of arachidonic acid (500 μM) in PRP samples containing each active derivative (100 μM) under stir conditions. After five minutes the suspensions were mixed with indomethacin (3 μL, 10 mM) stop-solution. By sonication for 1 min, platelets were disrupted and purification step were performed according to the manufacturer’s instructions (Cayman Chemical Co.). Plasma levels of PGE_2_ were measured in duplicate by competitive immunoassay using commercially available kits for EIA (PGE_2_ EIA Kit, Cayman Chemical Co.) according to the manufacturer’s instructions. A threshold of 25.75 ng/mL was defined as control plasma levels of PGE_2_ after platelet disrupture.

#### 3.2.5. Hemolysis Assay

Healthy erythrocytes were washed three times with PBS (pH 7.4) by centrifugation and suspended in the same buffer. All derivatives were incubated with the erythrocyte suspension for 3 h at 37 °C. The release of hemoglobin was determined by monitoring the optical density of the supernatant at 540 nm. The experiments were performed in triplicate and complete hemolysis (positive control) was determined using 1% Triton X-100. Hemolysis less than 10% indicated good hemocompatibility and non-toxicity of the molecules tested [48,79,80].

#### 3.2.6. Reverse Mutagenesis to Histidine Prototrophy (Ames Test)—“Spot Test”

This assay was performed as described by Maron and Ames [81], using the histidine *Salmonella typhimurium* auxotroph mutant strains TA97, TA98, TA100 and the wild type strain TA102 (Table 5). Each assay was conducted in duplicate and the results obtained show a comparison between the thiourea derivatives and the positive control 4-NQO. The negative results indicated that the thiourea derivatives have no mutagenic properties [81,82].

**Table 5:** Strains of *Salmonella typhimurium* used in the reverse mutagenesis to histidine prototrophy (Ames test)—“Spot test”.

Designations	Relevant Genotype
TA97	*his*D6610/ *his*O1242—Δ*uvr*B *rfa* pKM101 *(ampR*)
TA98	*his*D3052—Δ*uvr*B *rfa* pKM101(*amp*R)
TA100	*his*G46—Δ*uvr*B *rfa* pKM101 (*amp*R)
TA102	*his*G428-wild type *rfa*pKM101(*amp*R) pAQ1 (*tet*R)

#### 3.2.7. SOS Chromotest—“Spot Test”

The SOS chromotest (spot test) was performed according to Quillardet and Hofnung [53], using *Escherichia coli* (Table 6) strains PQ35 and PQ37. One hundred microliters of an overnight culture of the *E. coli* strains are diluted in 5 mL of LB medium and the culture is incubated at 37 °C in a gyratory incubator up to a concentration of 2 × 10^8^ bacteria/mL. Fractions of 0.1 mL of the culture are then distributed into test tube with top agar, and the mixture is poured immediately on M63 medium plate. A sample of 10 µL of the thiourea derivatives is spotted onto the center of the plate. After overnight incubation at 37 °C, the presence of a blue ring around a zone of inhibition indicates genotoxic activity. Each assay was conducted in triplicate and the results obtained show a comparison between the thiourea derivatives and the positive control 4-NQO.

**Table 6:** Strains of *Escherichia coli* used in SOS chromotest—“Spot test”.

Designations	Critical Markers	Other Markers
PQ35	*sfiA*::Mud(Ap*lac*) *cts* *lac*ΔU169 *mal*^+^, *uvr*^+^, *galEgalY*, PhoC, *rfa*	Same markers as GC4436 *rpoB*
PQ37	*sfiA*::Mud(Ap*lac*) *cts* *lac*ΔU169 *mal*^+^, *uvrA*, *galEgalY*, PhoC, *rfa*	Same markers as GC4436 *rpoB*

### 3.3. Molecular Modeling

#### SAR and Docking Studies

All molecular computations were performed using SPARTAN'10 (Wavefunction Inc. Irvine, CA, USA) as described elsewhere [77,83,84,85]. Briefly, the structures were optimized to a local minimum and the equilibrium geometry was obtained in a vacuum using RM1 semi empirical methods. Subsequently, molecules were submitted to a single-point energy *ab initio* calculation, at the 6-31G** level, to calculate some stereoelectronic properties and to perform SAR studies. Thus, we calculated HOMO and LUMO energies and isosurface density, molecular weight, molecular surface area, polar surface area, dipole moment, lipophilicity and electrostatic potential maps for all compounds best conformation.

Docking studies were carried on using the ovine COX-1 in complex with indomethacin (PDB ID: 2OYE) crystal structure and a TXS model recently publishes [44]. Briefly, the three-dimensional grid was set over both COX-1 and TXS active site in a 60 × 60 × 60 Å grid box with 0.171 Å spacing between points. Ligand torsions were set using the maximum freedom while the protein structure was set as rigid. Grid parameters were generated using the software Autogrid 4.0 available in AutoDock Tools (TSRI^©^, La Jolla, CA, USA), molecular docking was performed using the software AutoDock 4.0 with Lamarkian genetic algorithm through 50 generations. Redocking of indomethacin resulted in a stable model with an overall RMSD of 1.7 Å to the crystallographic pose, prompting the usage of this method for further studies with thiourea derivatives [86,87].

## 4. Conclusions

In this work we present a series of *N,N'*-disubstituted thioureas **3a**–**q** that were designed and synthesized in high yields (82%–97%) as non-anionic antiplatelet agents against the arachidonic acid platelet aggregation pathway. The most active antiplatelet agents (compounds **3d**, **3i**, **3m** and **3p**) were able to reduce both PGE_2_ and TXB_2_ production in human platelets, suggesting a direct inhibition of COX-1. Structural features such as hydrophobicity, chain length and presence of HBA groups were observed as important characteristics for the proposed mechanism of action. This series of compounds shows low mutagenic and genotoxic profiles according to Ames test and SOS chromotest, and good hemocompatibility toward healthy human erythrocyte, which reinforce their promising lead profile as antiplatelet agents for further *in vivo* experimental investigations.

## Supplementary Materials

Supplementary materials can be accessed at: http://www.mdpi.com/1420-3049/20/04/7174/s1.
