# Supplementary material for: Synthesis and Antiplatelet Activity of Antithrombotic Thiourea Compounds: Biological and Structure-Activity Relationship Studies
Source: Molecules. 2015 Apr 20;20(4):7174–200. doi: 10.3390/molecules20047174 (PMC6272548; doi:10.3390/molecules20047174)
Supplement: Supplementary file 1 [file molecules-20-07174-s001.pdf]

Supporting Information

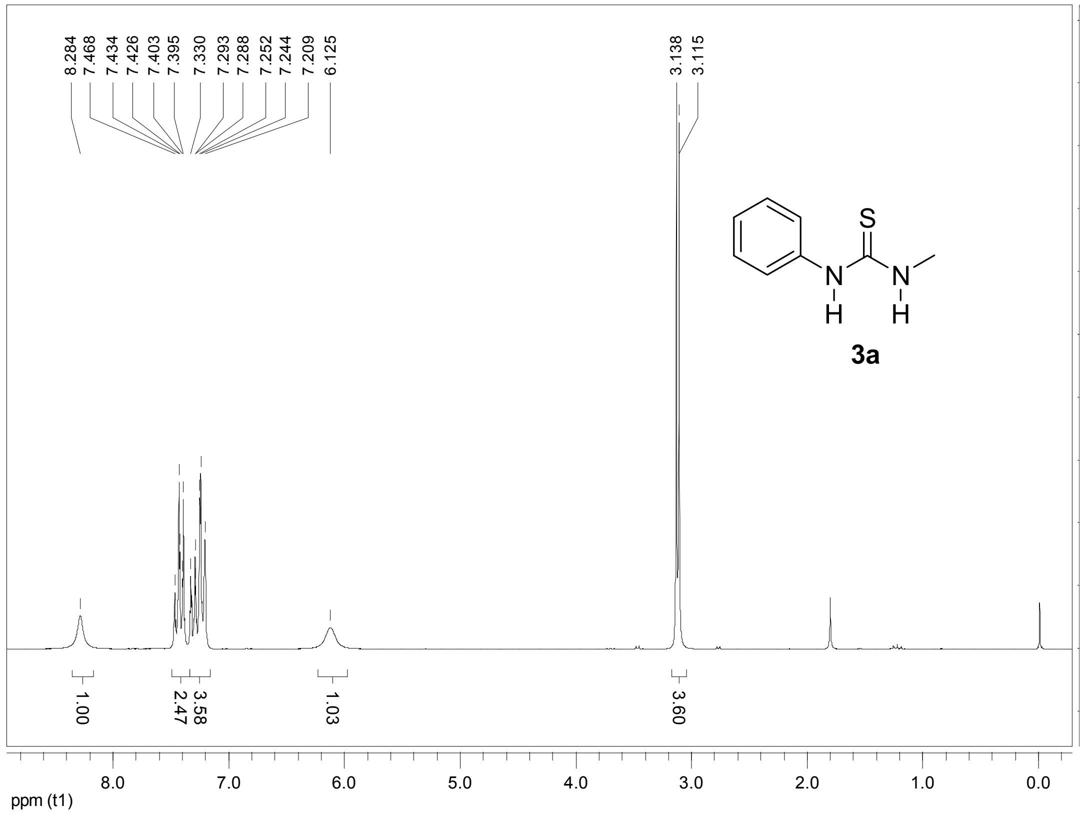

(**A**)

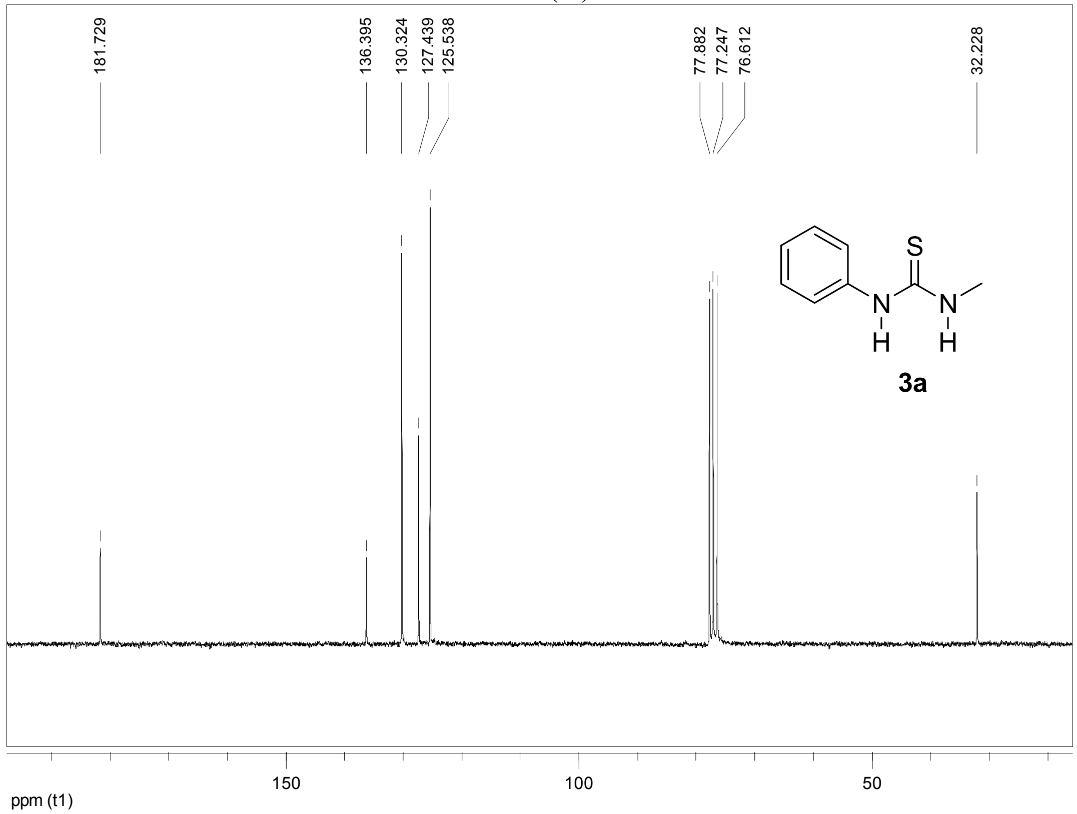

(**B**)

Figure S1. ^{1}H-NMR spectrum (**A**) and ^{13}C-NMR spectrum (**B**) of thiourea **3a**.

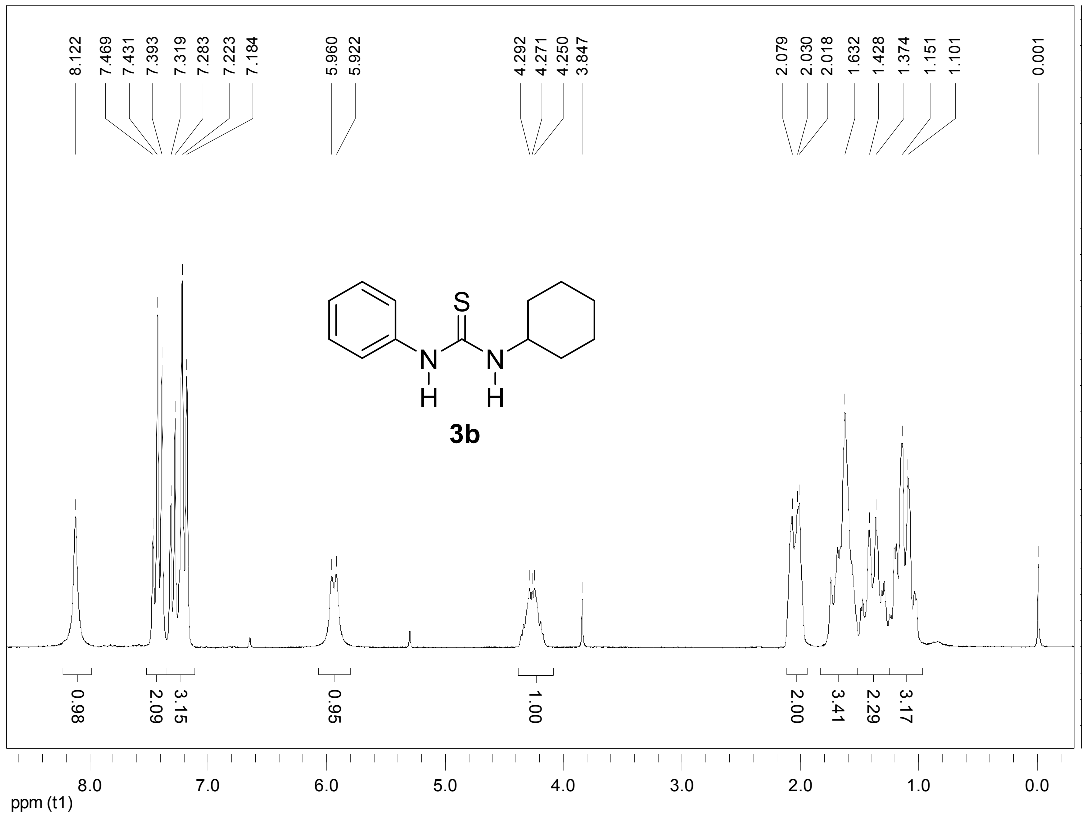

(A)

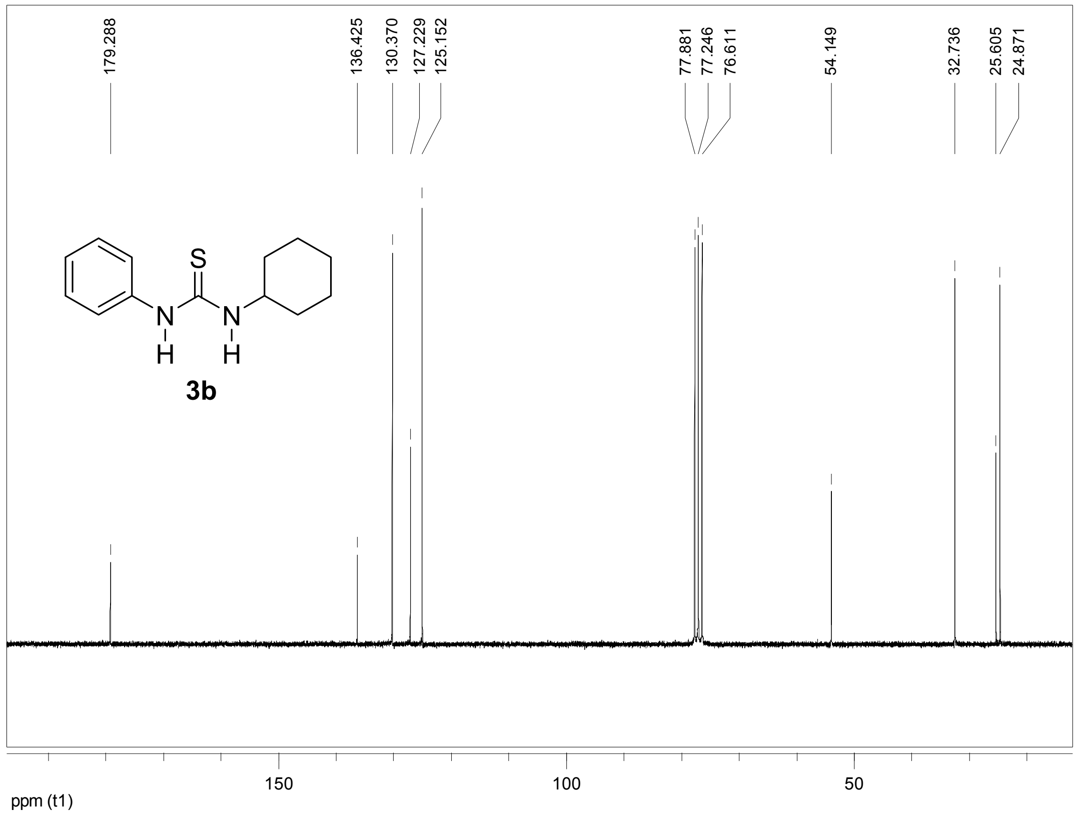

(B)

Figure S2. ^{1}H-NMR spectrum (**A**) and ^{13}C-NMR spectrum (**B**) of thiourea **3b**.

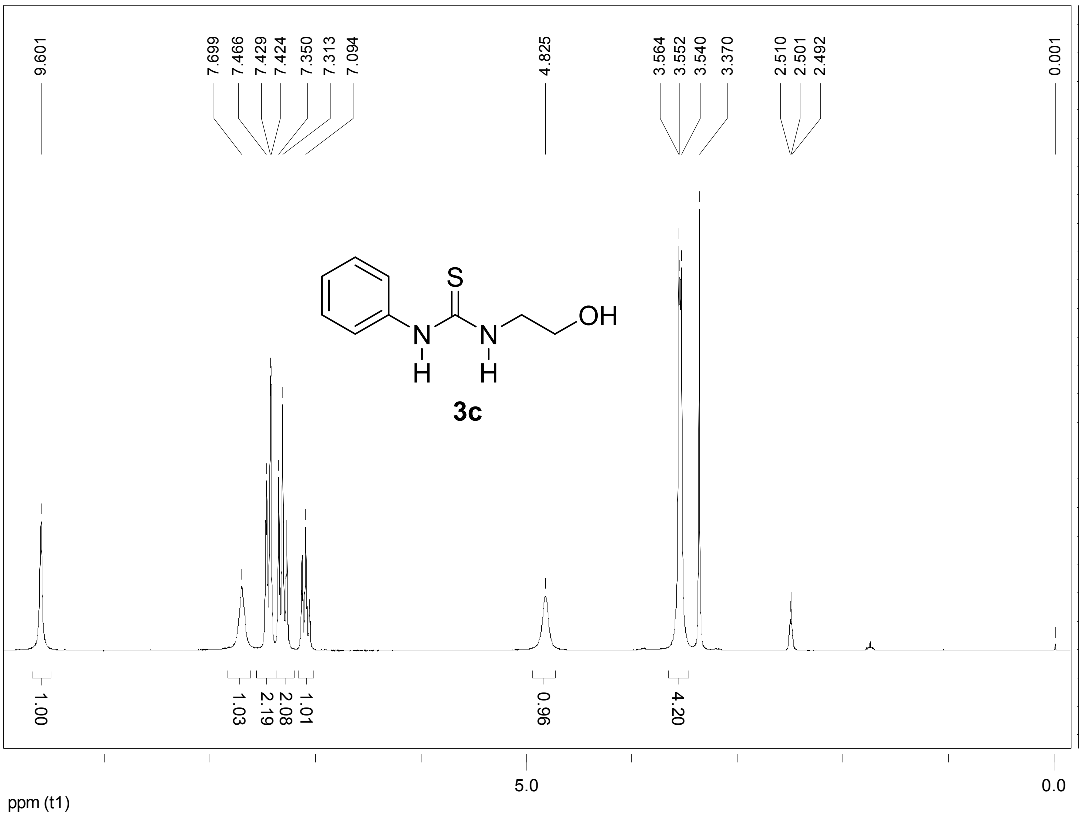

(**A**)

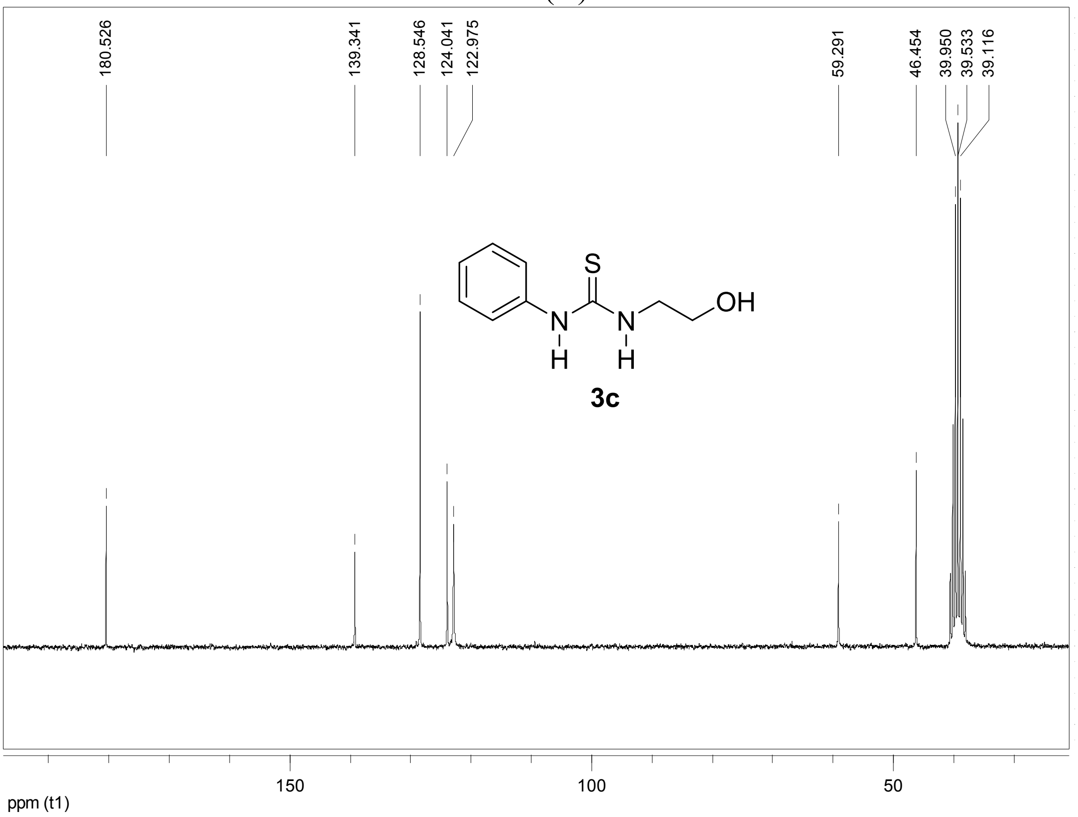

(**B**)

Figure S3. ^{1}H-NMR spectrum (**A**) and ^{13}C-NMR spectrum (**B**) of thiourea **3c**.

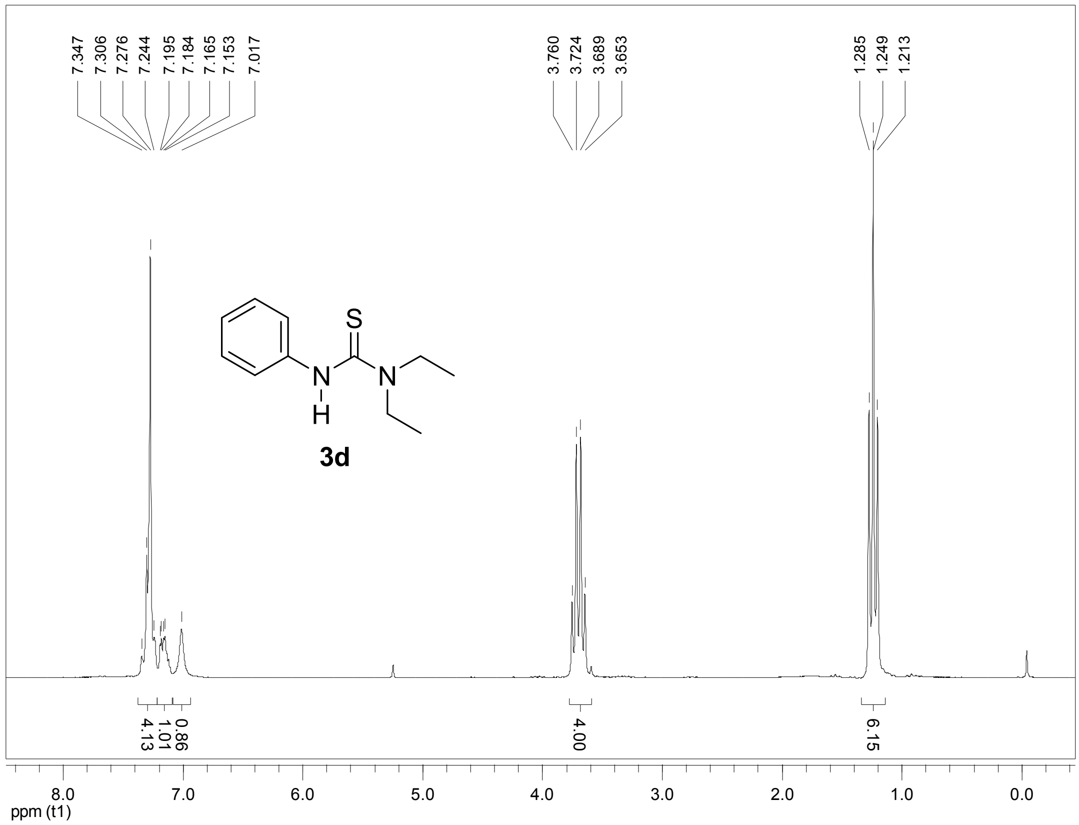

(**A**)

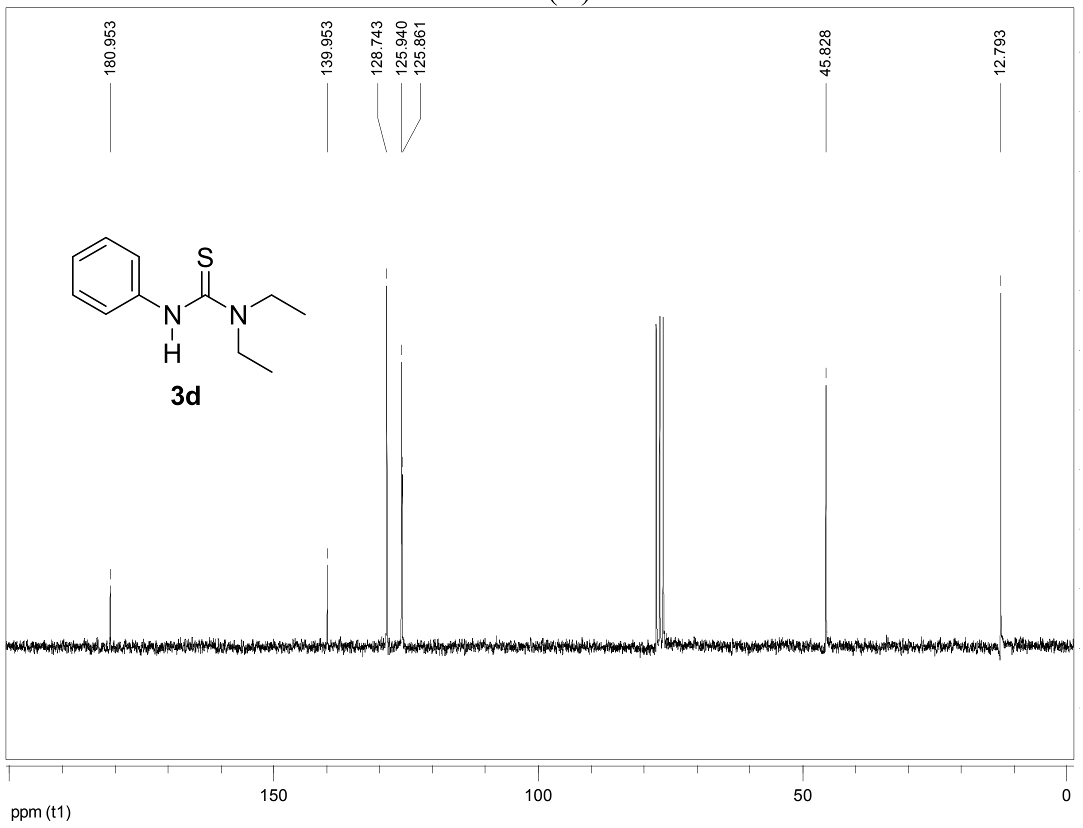

(**B**)

Figure S4. ^{1}H-NMR spectrum (**A**) and ^{13}C-NMR spectrum (**B**) of thiourea **3d**.

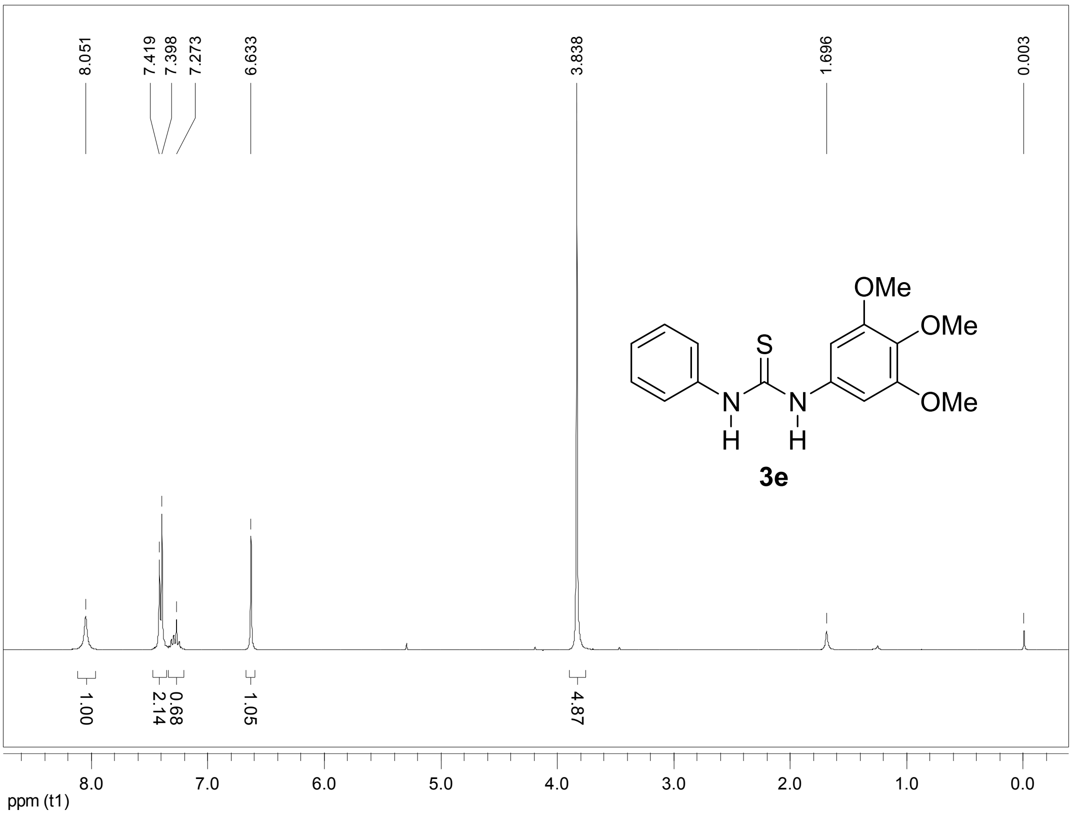

(**A**)

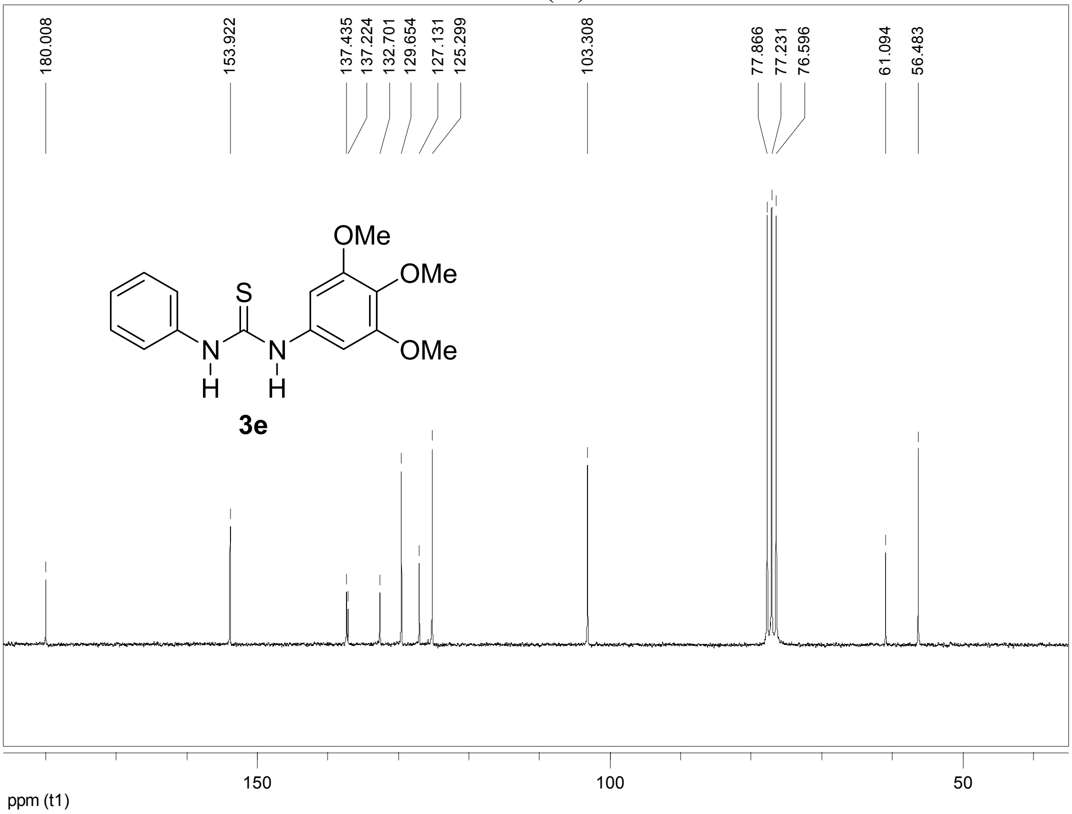

(**B**)

Figure S5. ^{1}H-NMR spectrum (**A**) and ^{13}C-NMR spectrum (**B**) of thiourea **3e**.

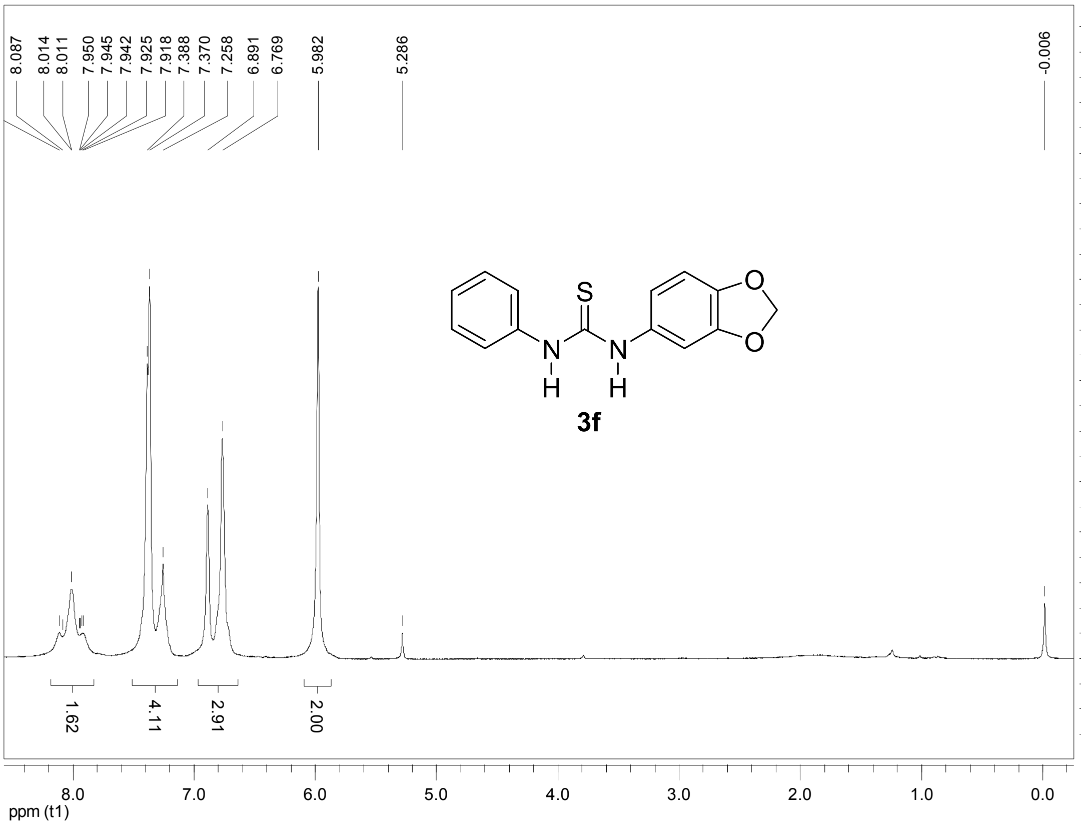

(**A**)

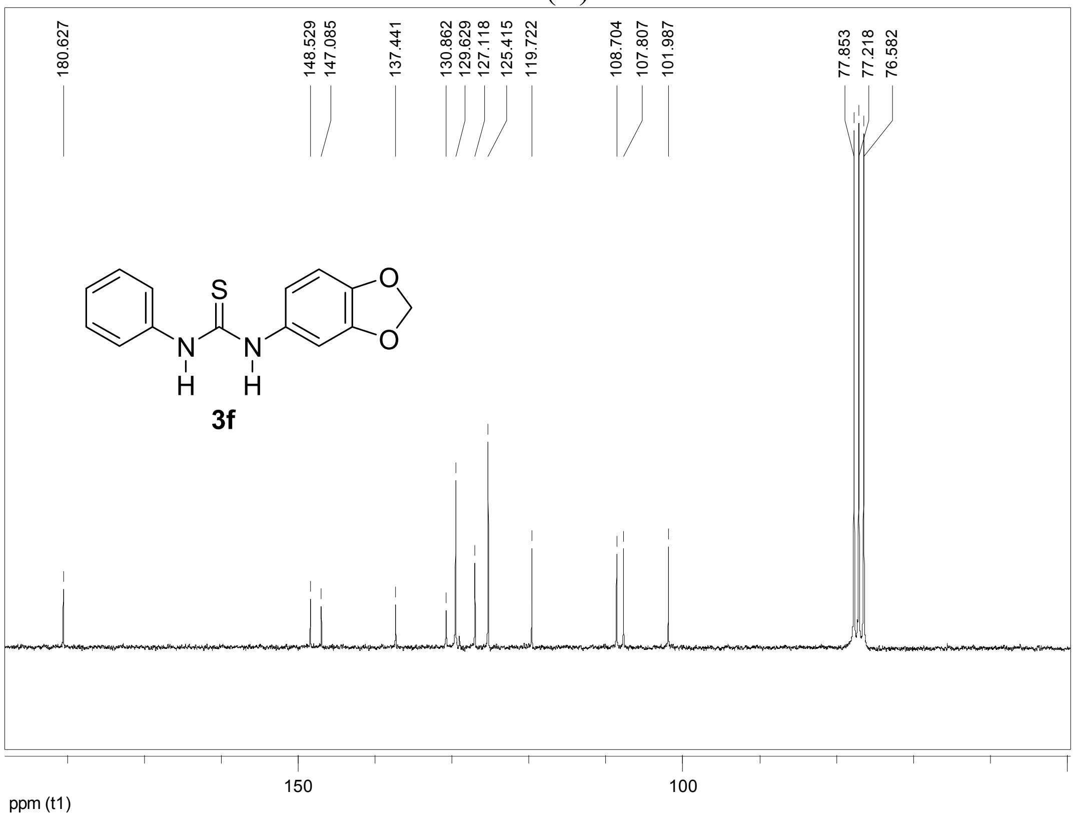

(**B**)

Figure S6. ^{1}H-NMR spectrum (**A**) and ^{13}C-NMR spectrum (**B**) of thiourea **3f**.

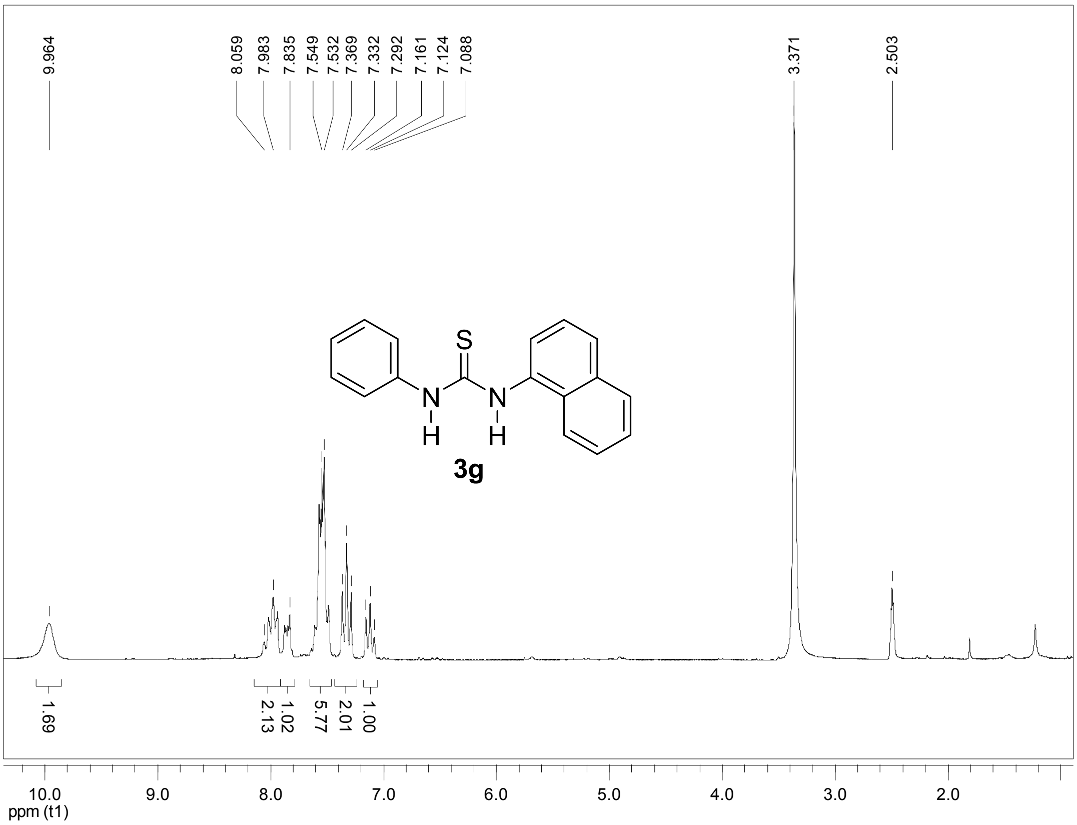

(**A**)

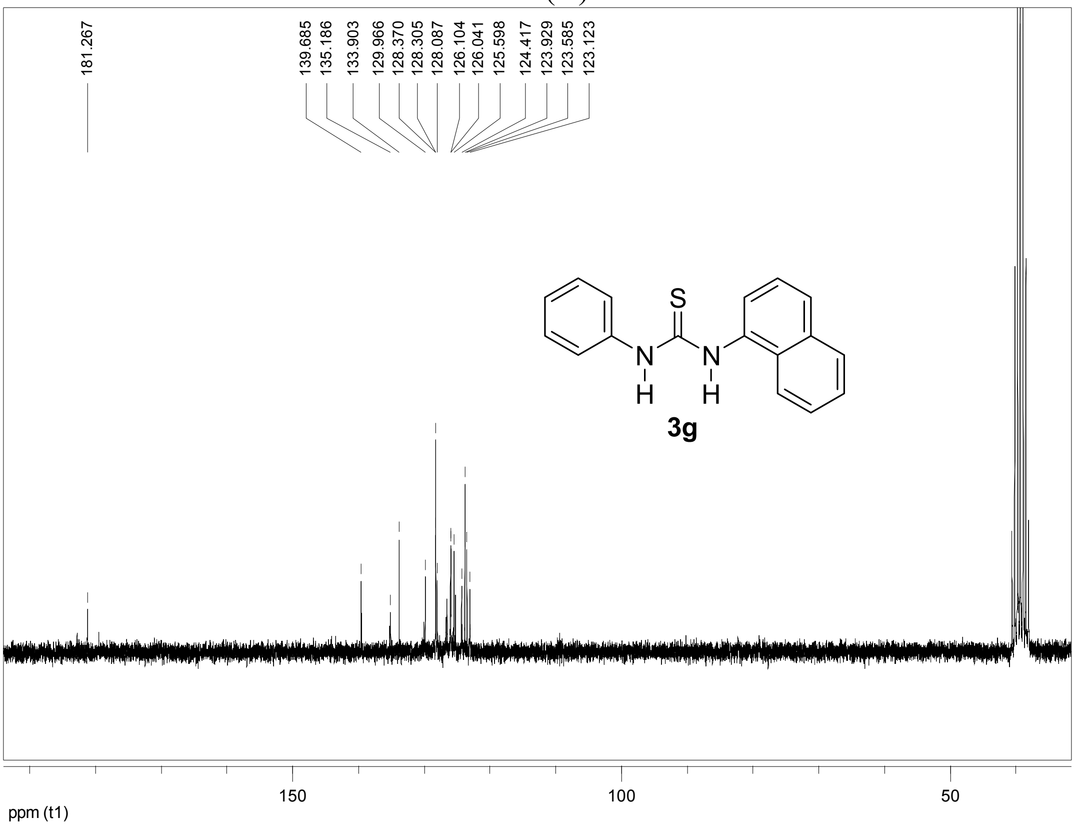

(**B**)

Figure S7. ^{1}H-NMR spectrum (**A**) and ^{13}C-NMR spectrum (**B**) of thiourea **3g**.

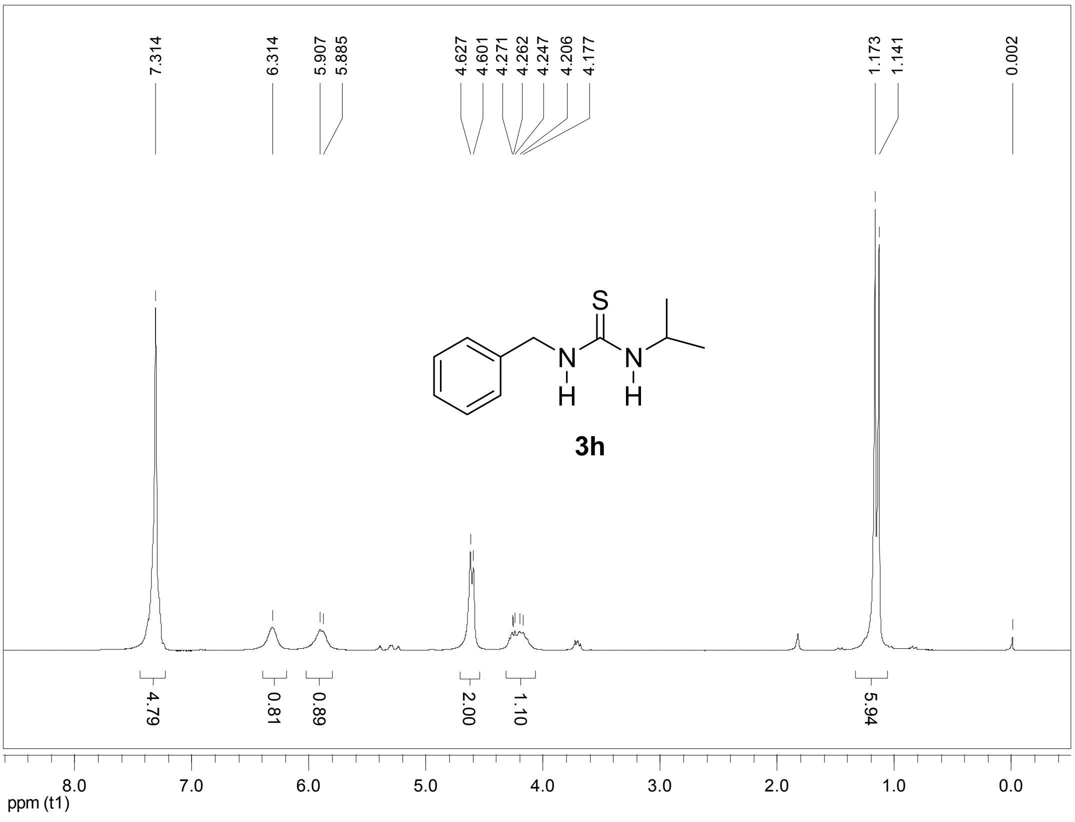

(**A**)

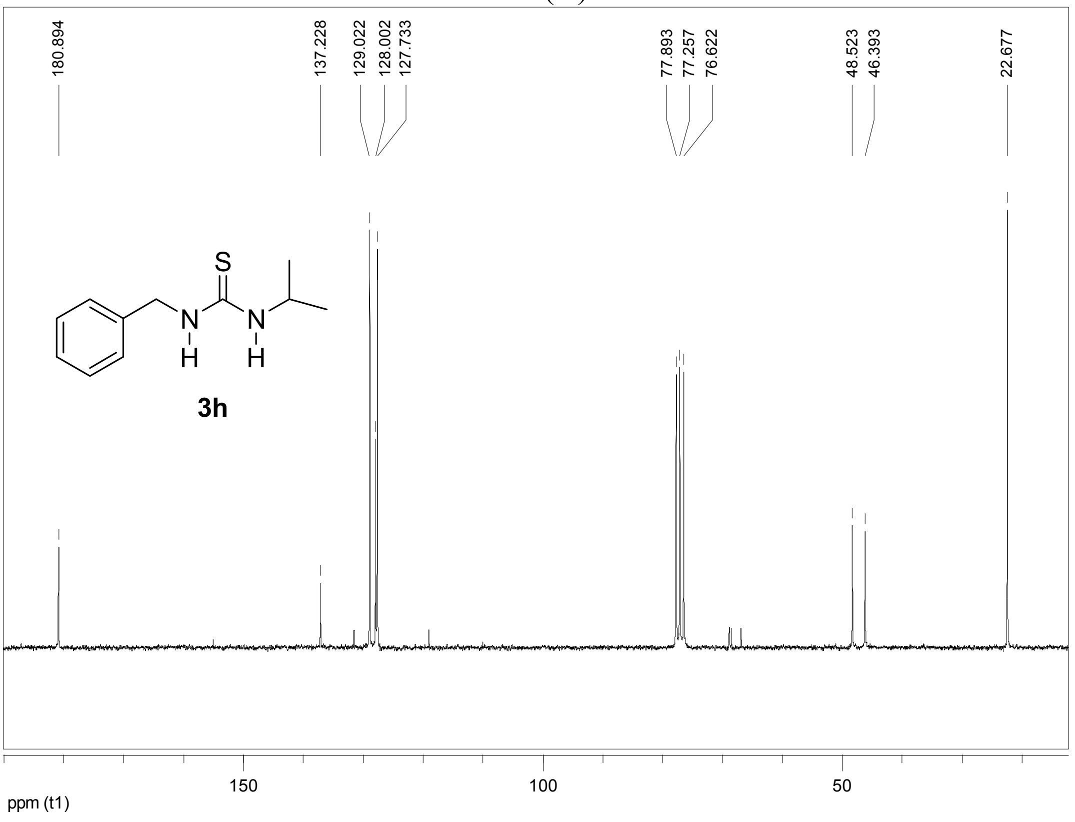

(**B**)

Figure S8. ^{1}H-NMR spectrum (**A**) and ^{13}C-NMR spectrum (**B**) of thiourea **3h**.

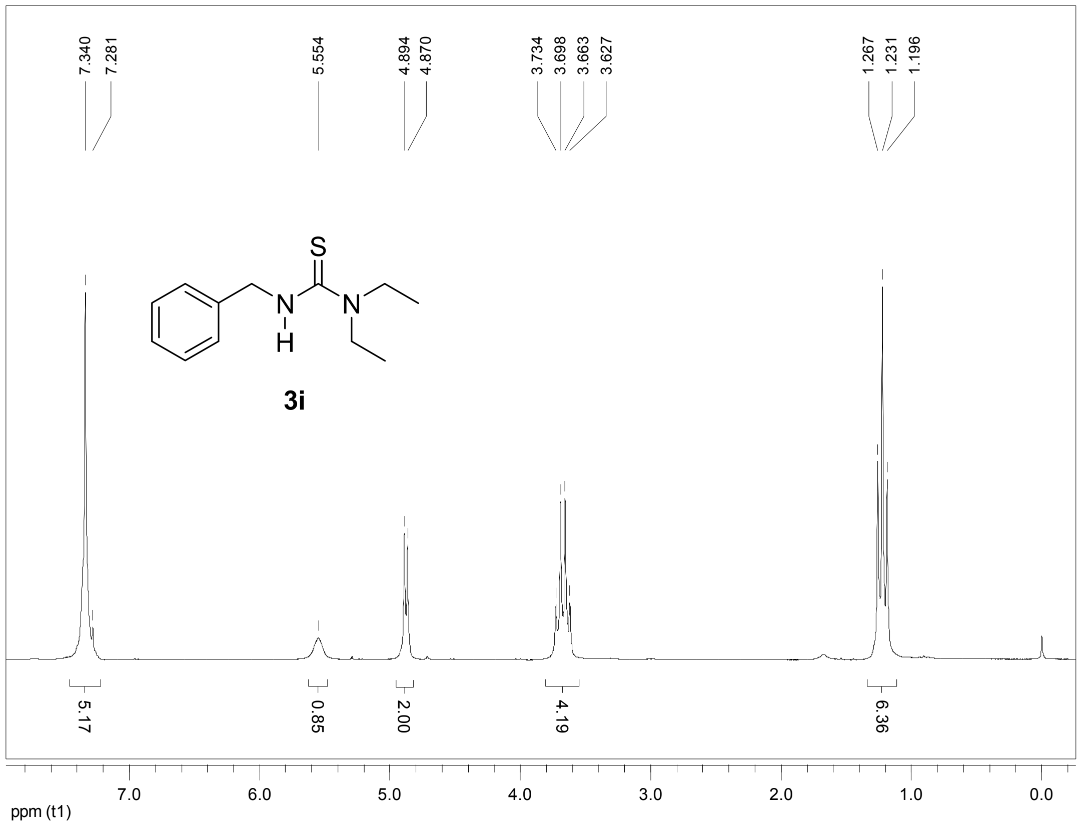

(**A**)

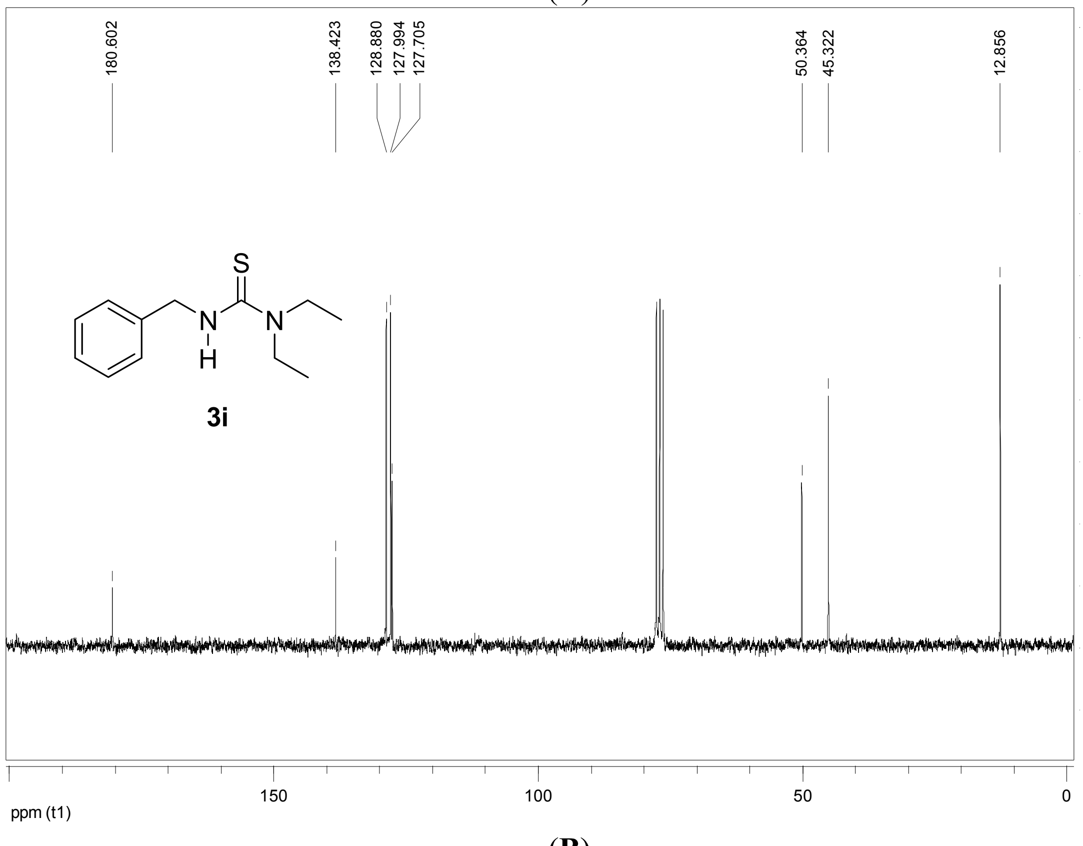

(**B**)

Figure S9. ^{1}H-NMR spectrum (**A**) and ^{13}C-NMR spectrum (**B**) of thiourea **3i**.

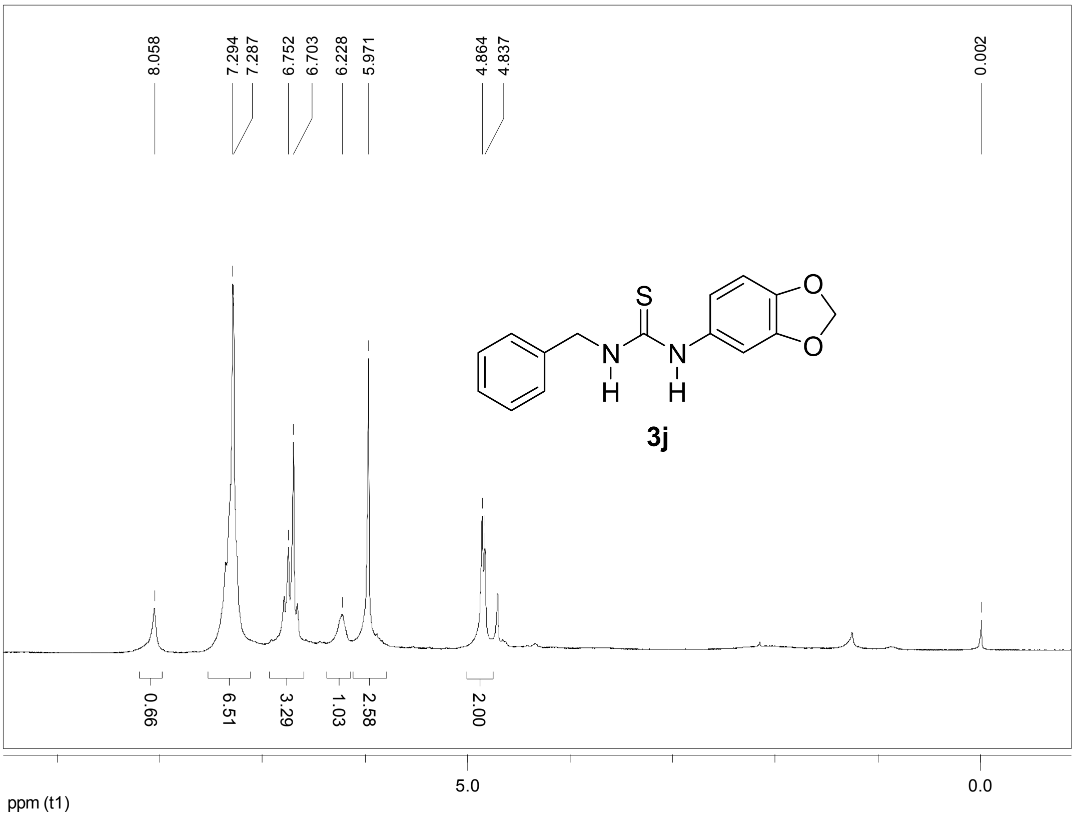

(**A**)

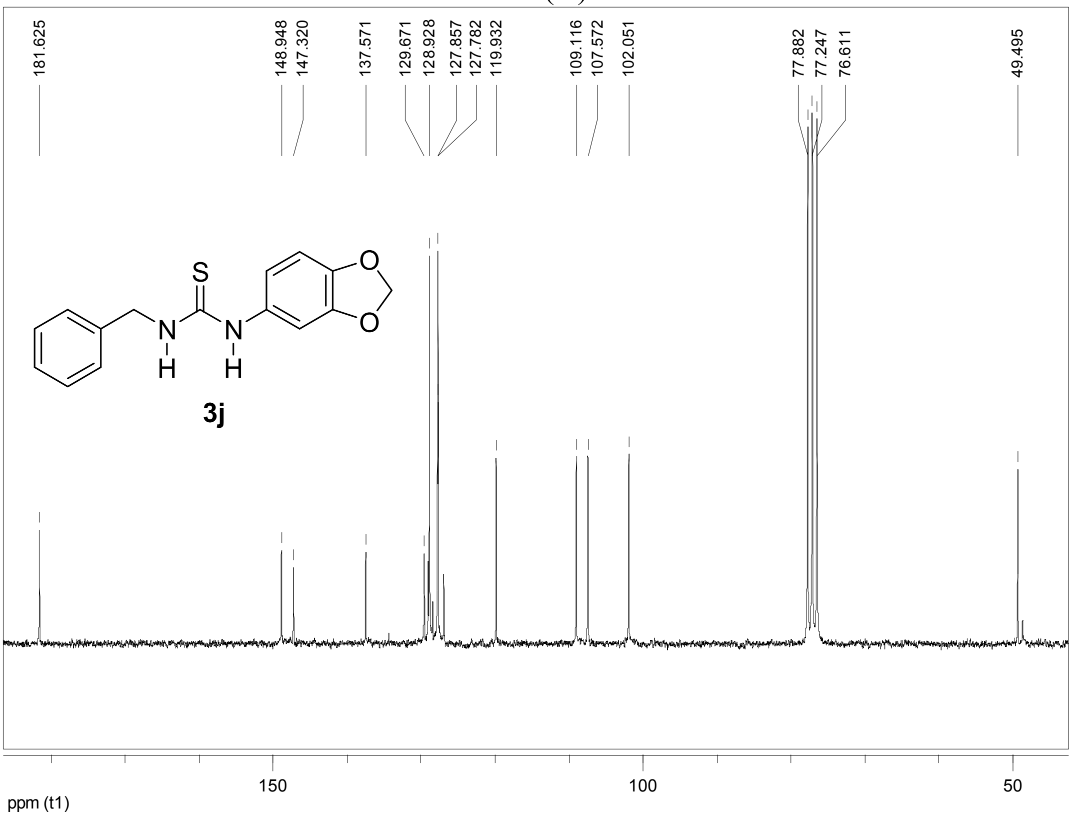

(**B**)

Figure S10. ^{1}H-NMR spectrum (**A**) and ^{13}C-NMR spectrum (**B**) of thiourea **3j**.

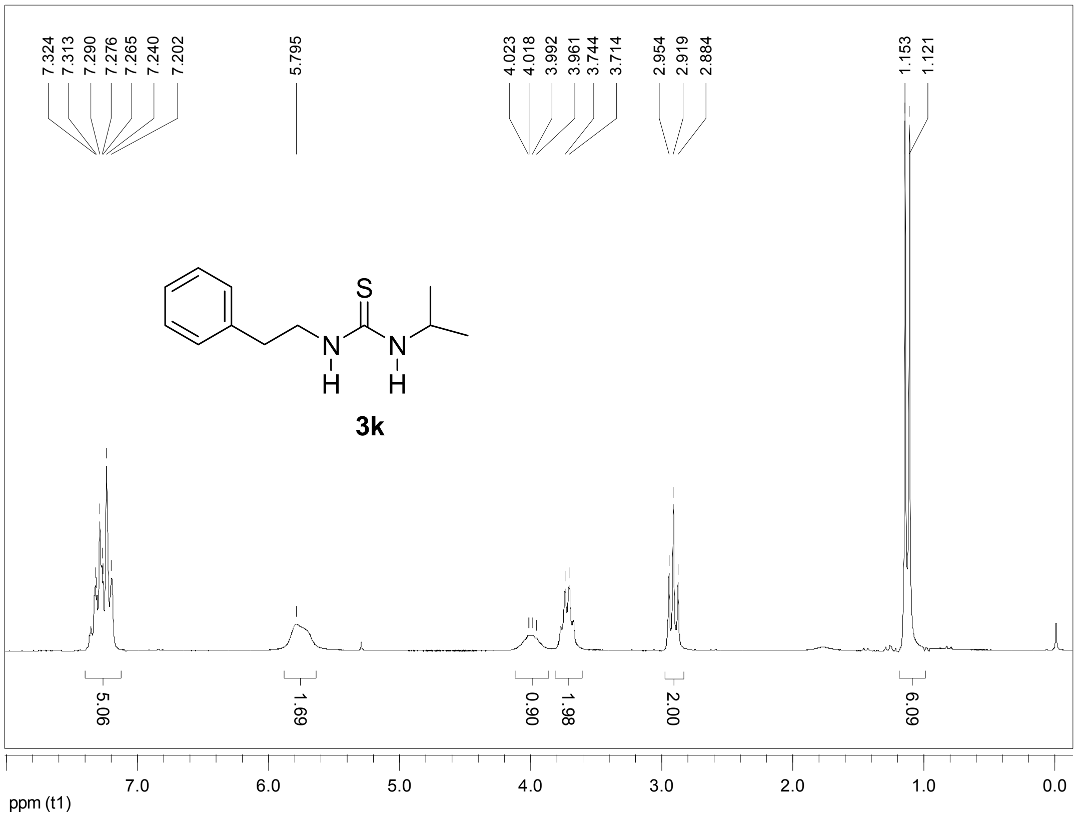

(**A**)

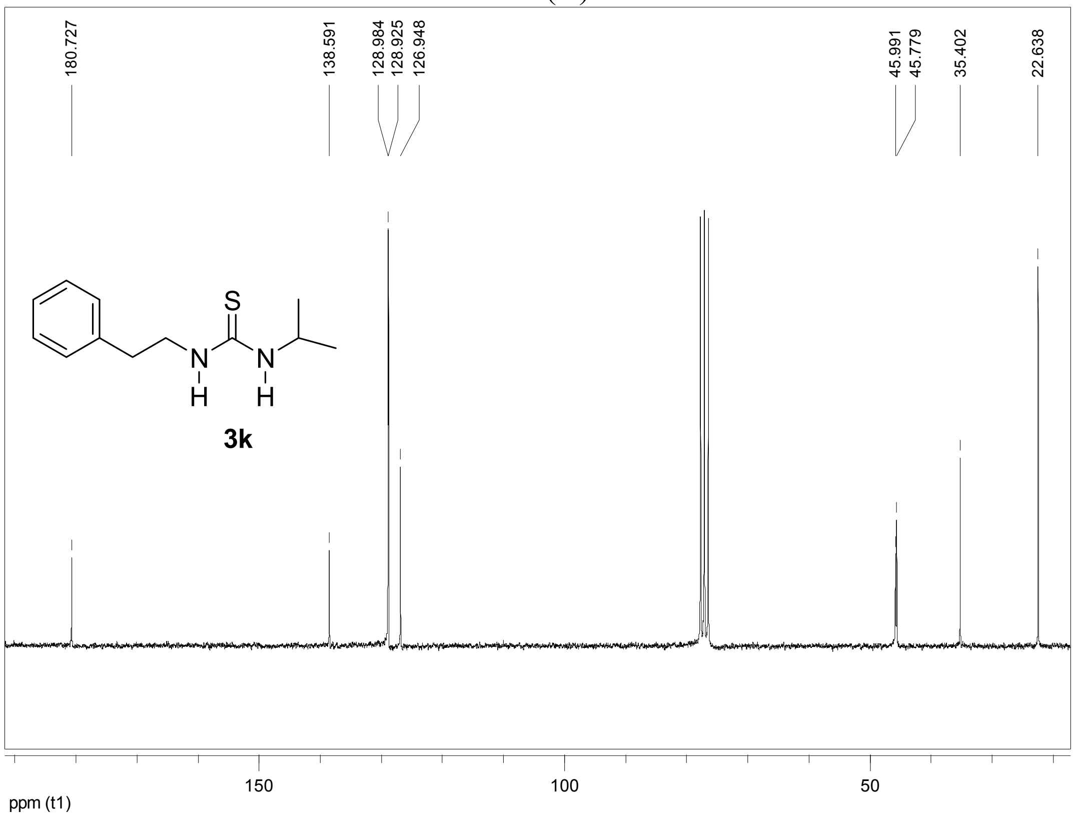

(**B**)

Figure S11. ^{1}H-NMR spectrum (**A**) and ^{13}C-NMR spectrum (**B**) of thiourea **3k**.

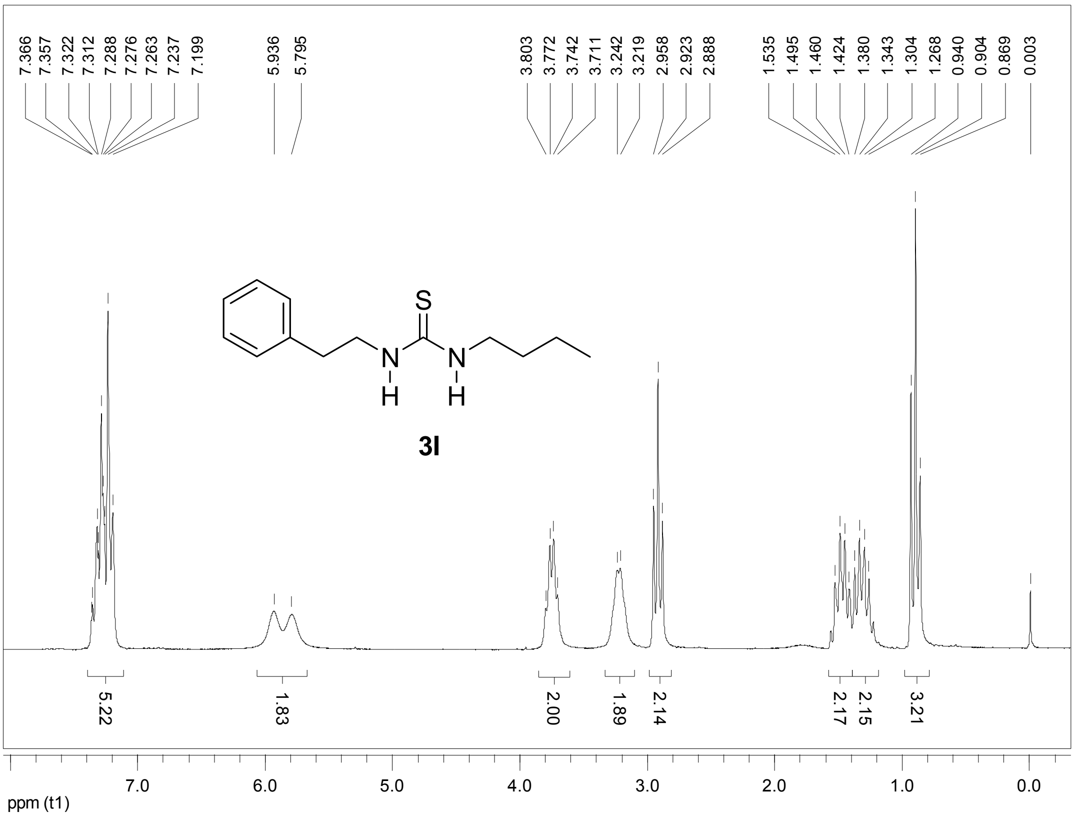

(**A**)

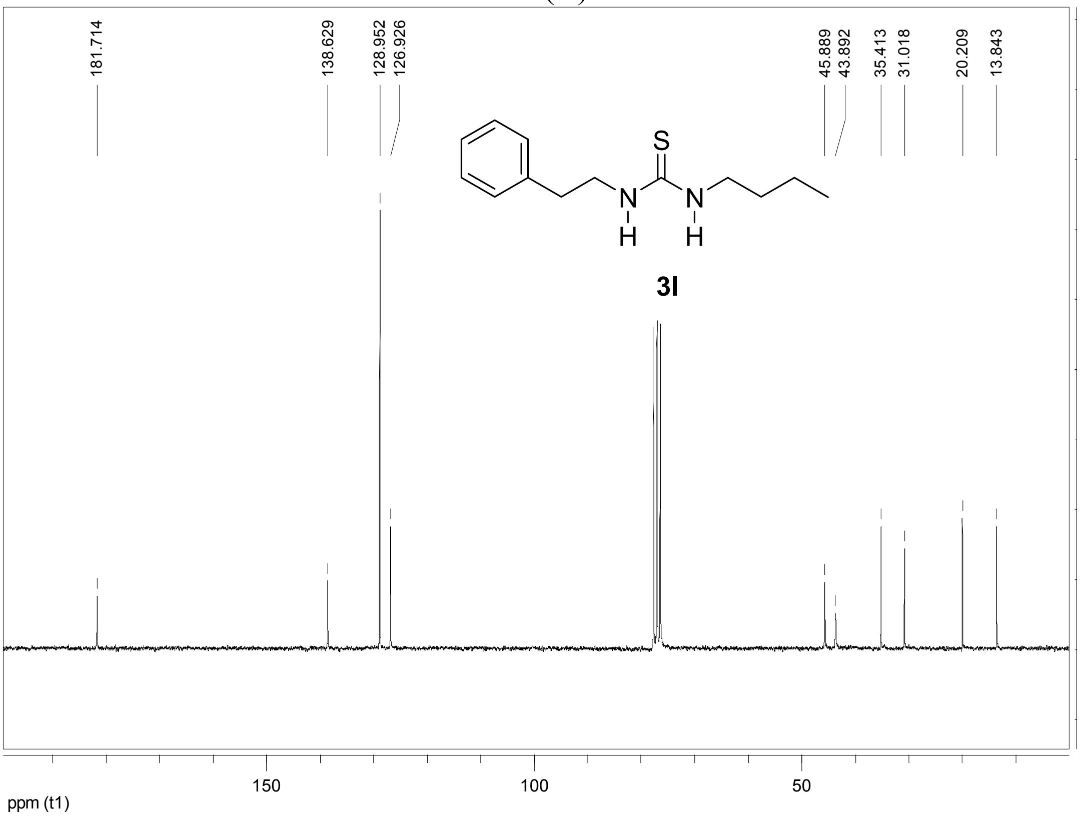

(**B**)

Figure S12. ^{1}H-NMR spectrum (**A**) and ^{13}C-NMR spectrum (**B**) of thiourea **3l**.

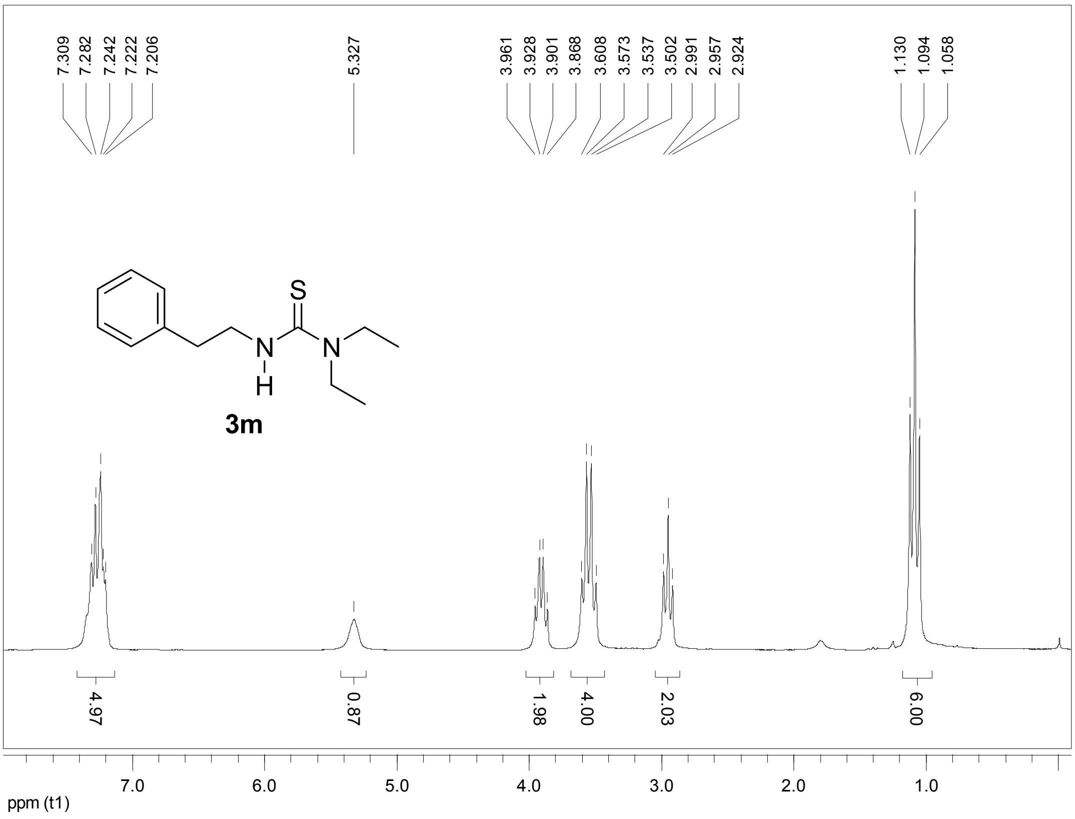

(**A**)

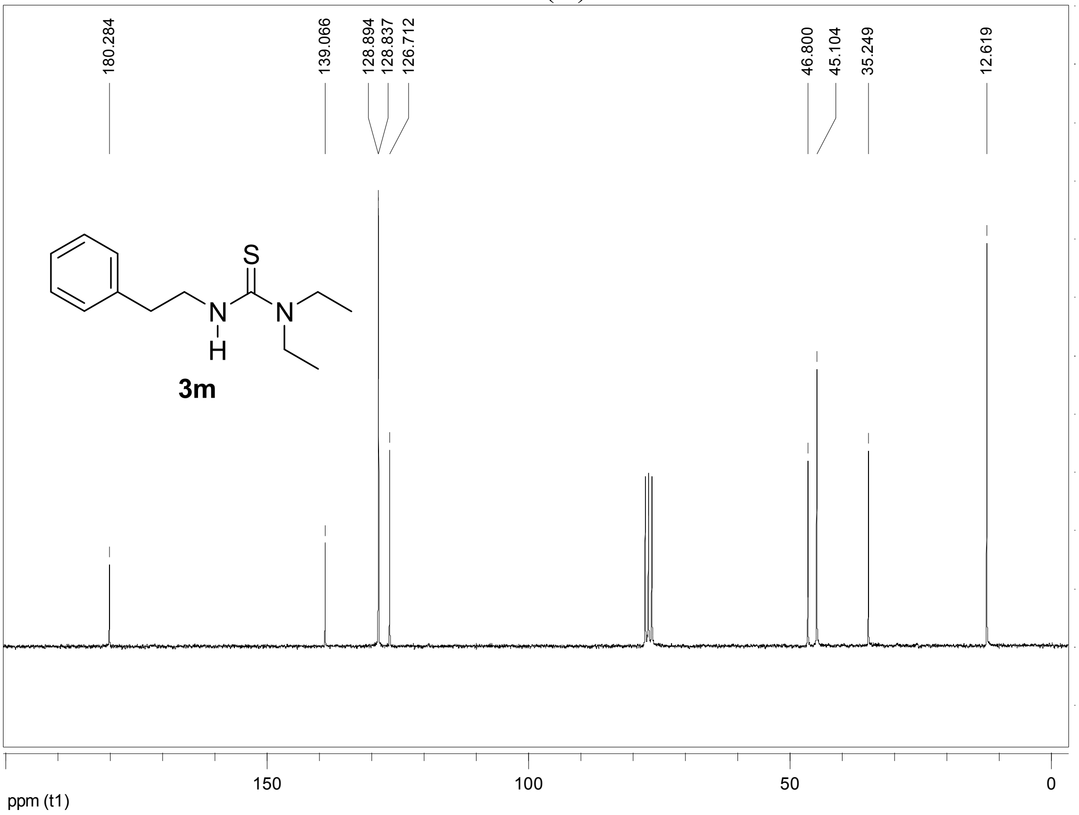

(**B**)

Figure S13. ^{1}H-NMR spectrum (**A**) and ^{13}C-NMR spectrum (**B**) of thiourea **3m**.

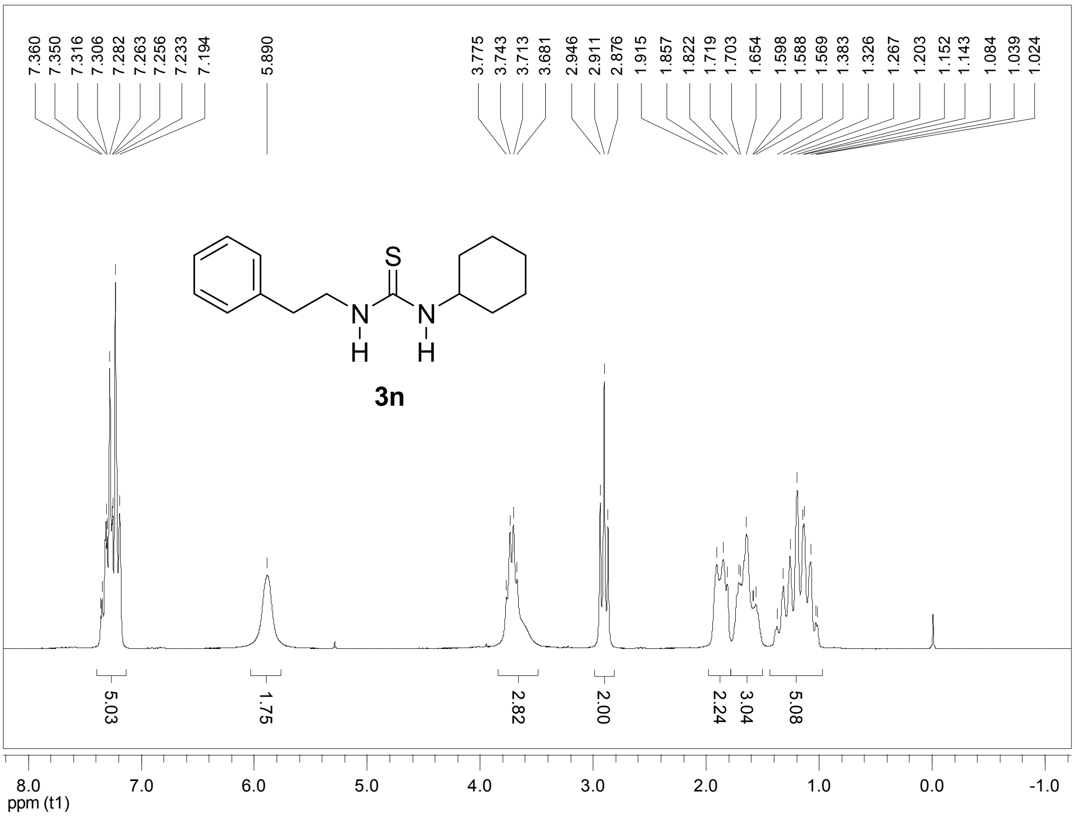

(**A**)

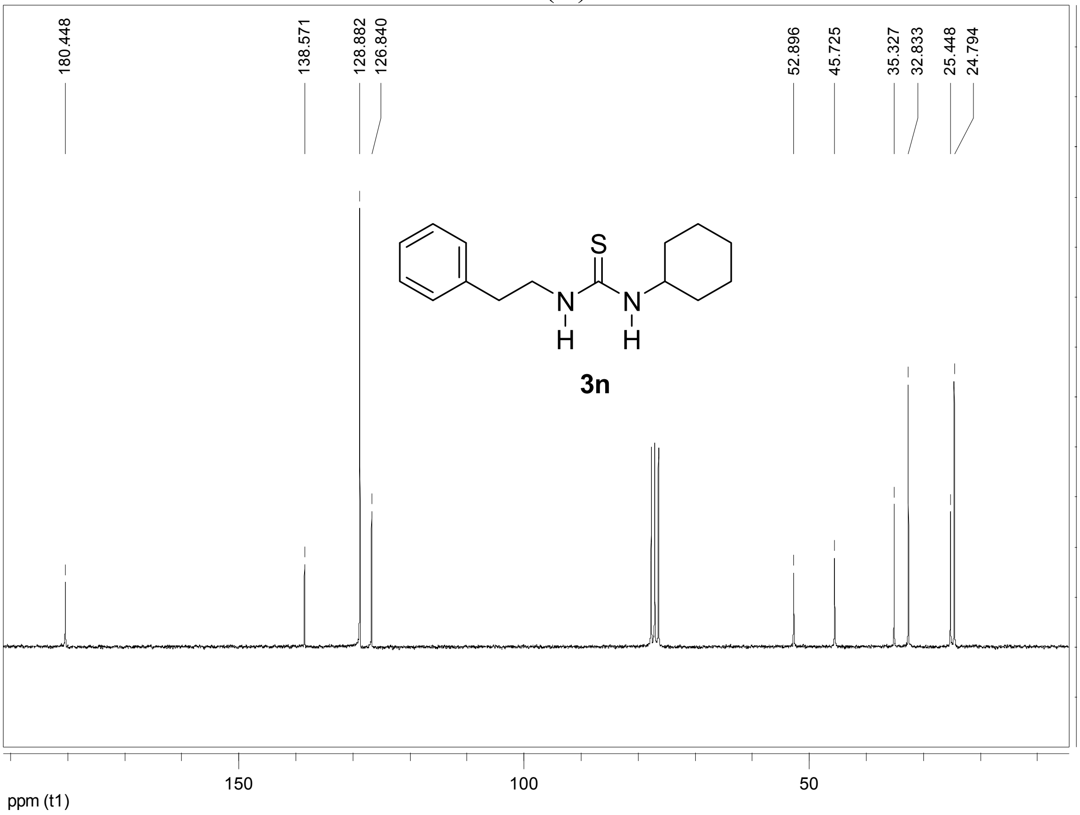

(**B**)

Figure S14. ^{1}H-NMR spectrum (**A**) and ^{13}C-NMR spectrum (**B**) of thiourea **3n**.

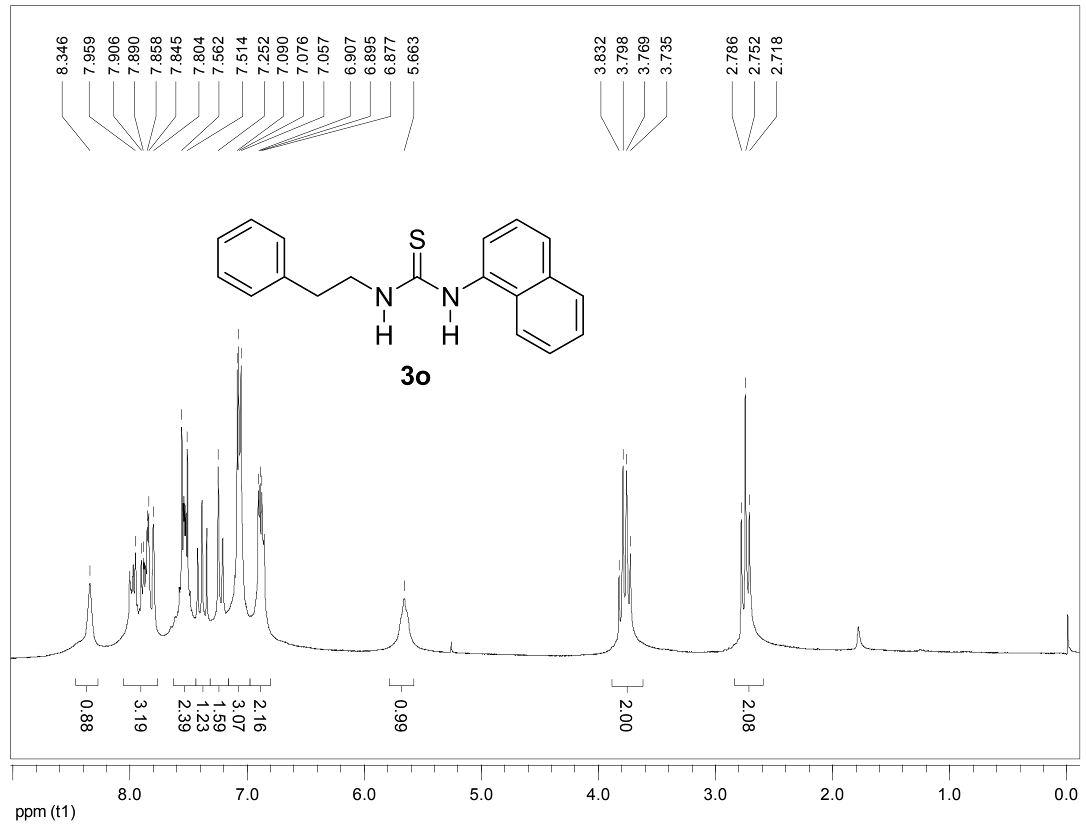

(**A**)

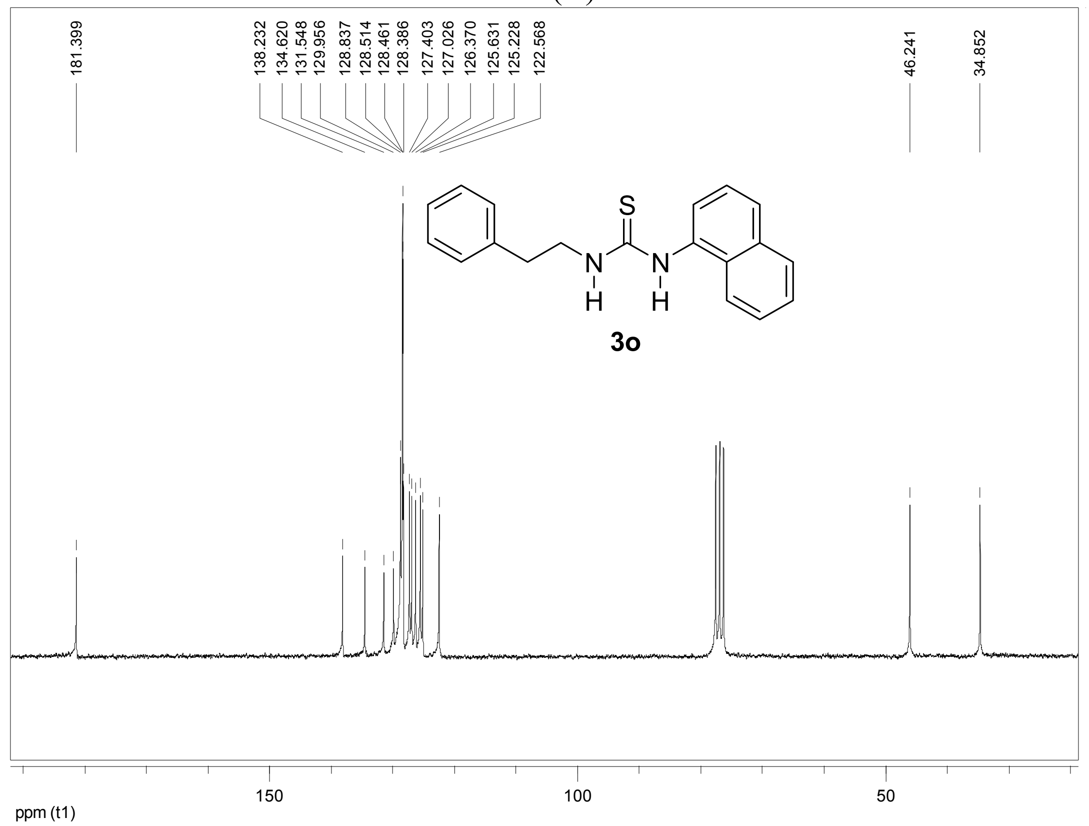

(**B**)

Figure S15. ^{1}H-NMR spectrum (**A**) and ^{13}C-NMR spectrum (**B**) of thiourea **3o**.

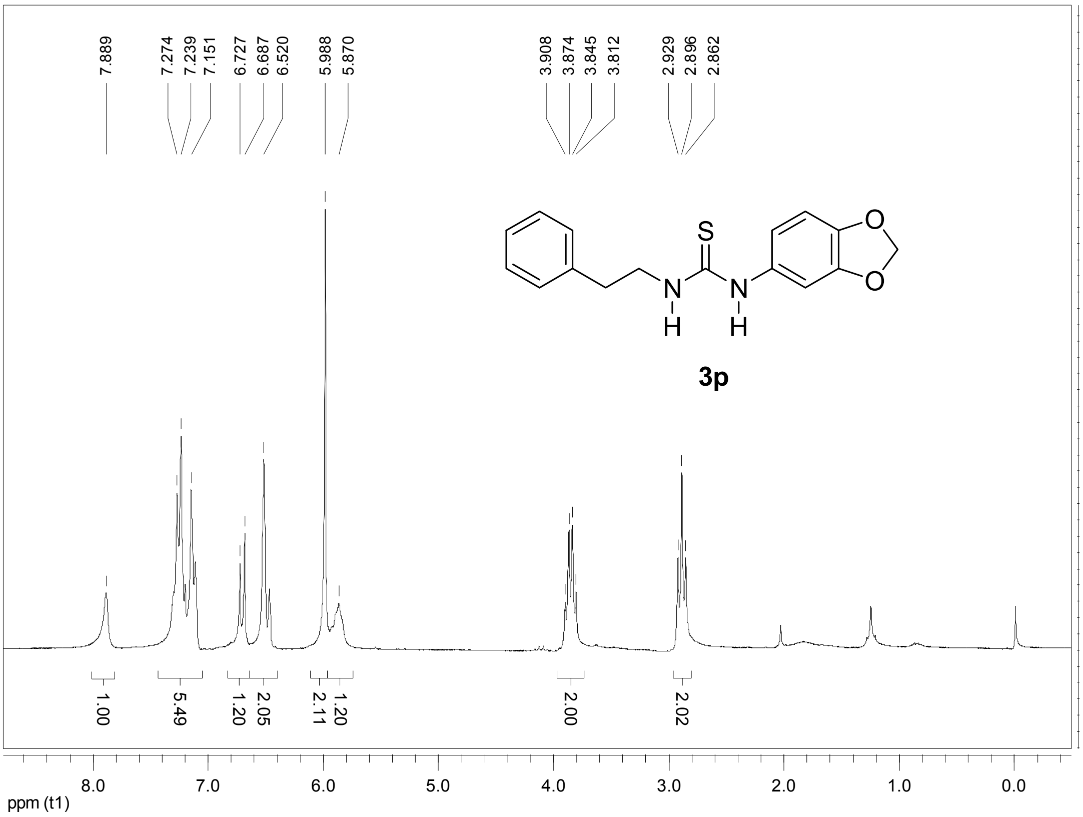

(**A**)

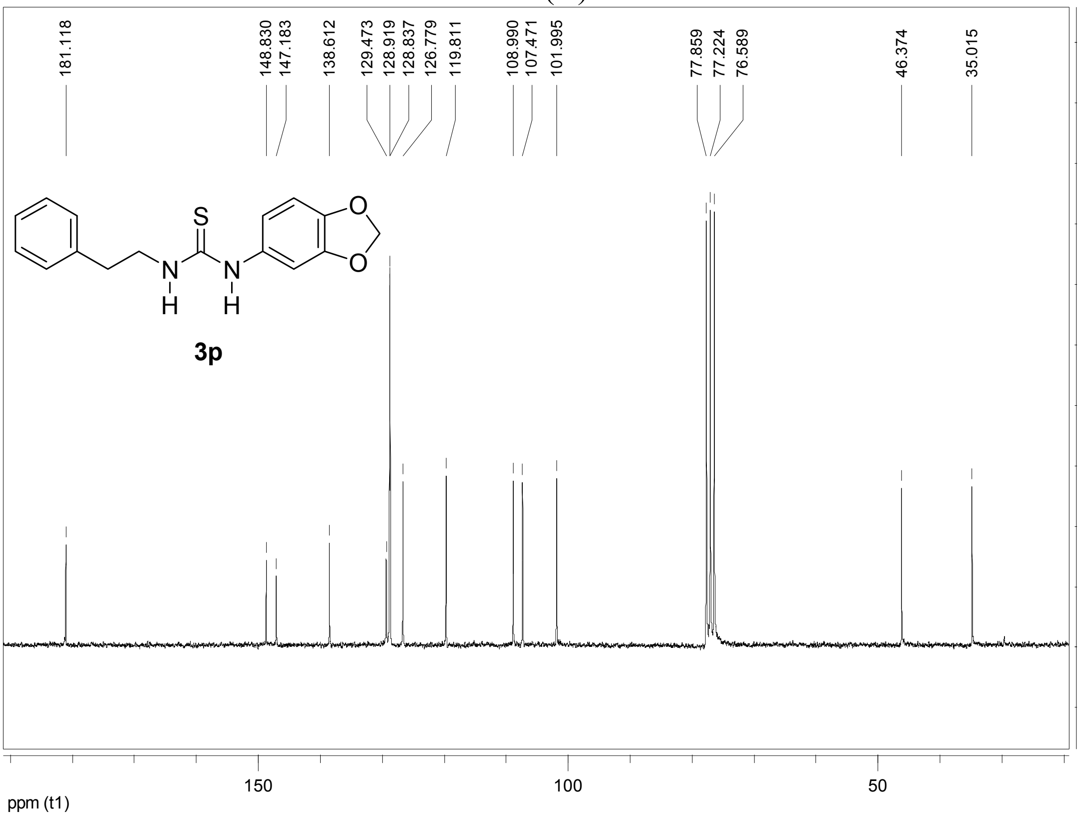

(**B**)

Figure S16. ^{1}H-NMR spectrum (**A**) and ^{13}C-NMR spectrum (**B**) of thiourea **3p**.

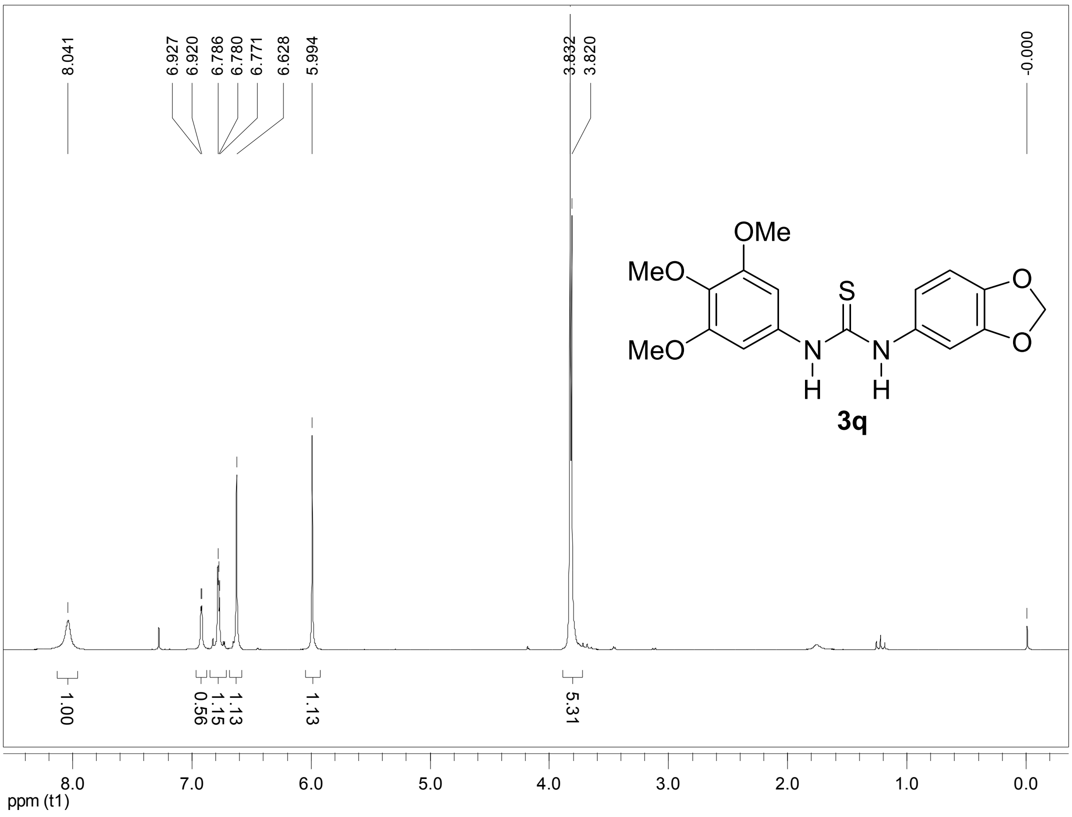

(**A**)

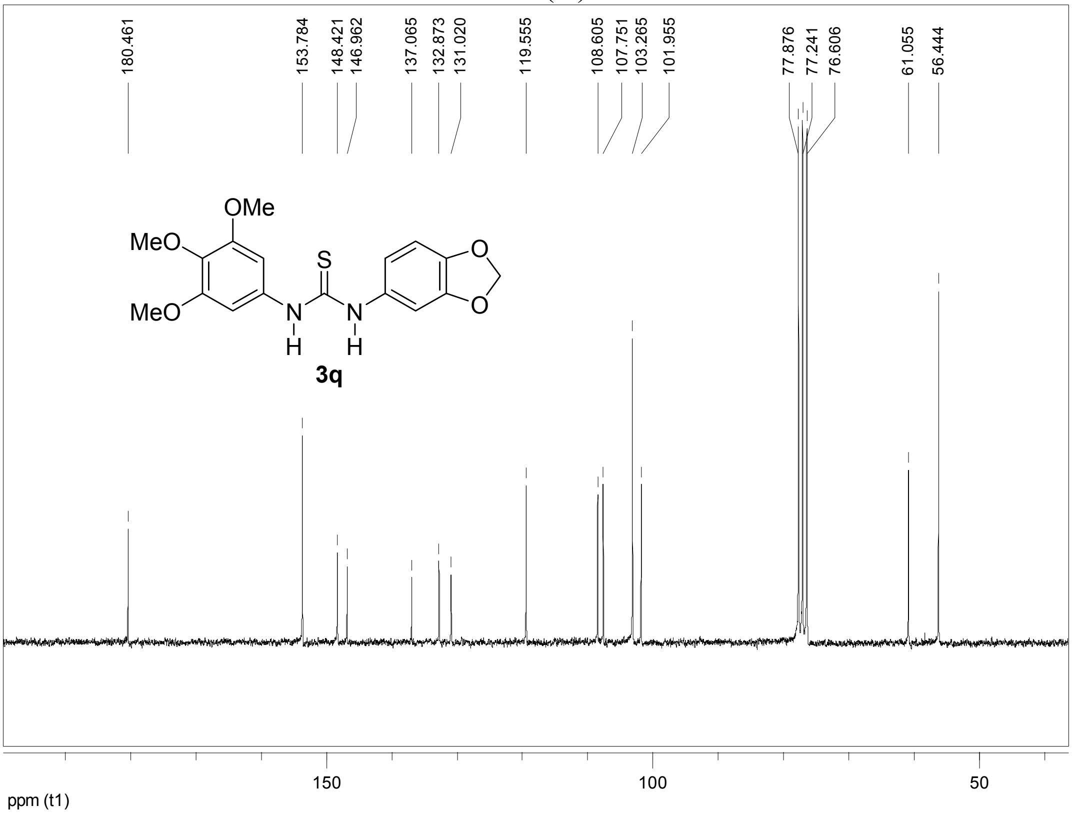

(**B**)

Figure S17. ^{1}H-NMR spectrum (**A**) and ^{13}C-NMR spectrum (**B**) of thiourea **3q**.
